# Pharmacological Mechanisms and Clinical Applications of Curcumin: Update

**DOI:** 10.14336/AD.2022.1101

**Published:** 2023-06-01

**Authors:** Min Hao, Yue Chu, Jingxuan Lei, Zhouhui Yao, Pingping Wang, Ziyan Chen, Kuilong Wang, Xianan Sang, Xin Han, Lu Wang, Gang Cao

**Affiliations:** ^1^School of Pharmaceutical Sciences, Zhejiang Chinese Medical University, Hangzhou 311402, China; ^2^School of Pharmacy, Zunyi Medical University, Zunyi 563006, China

**Keywords:** curcumin, pharmacological mechanism, bioavailability, clinical application

## Abstract

Curcumin, a well-known hydrophobic polyphenol extracted from the rhizomes of turmeric (Curcuma longa L.), has attracted great interest in the last ten years due to its multiple pharmacological activities. A growing body of evidence has manifested that curcumin has extensive pharmacological activities including anti-inflammatory, anti-oxygenation, lipid regulation, antiviral, and anticancer with hypotoxicity and minor adverse reactions. However, the disadvantages of low bioavailability, short half-life in plasma, low drug concentration in blood, and poor oral absorption severely limited the clinical application of curcumin. Pharmaceutical researchers have carried out plenty of dosage form transformations to improve the druggability of curcumin and have achieved remarkable results. Therefore, the objective of this review summarizes the pharmacological research progress, problems in clinical application and the improvement methods of curcumin’s druggability. By reviewing the latest research progress of curcumin, we believe that curcumin has a broad clinical application prospect for its wide range of pharmacological activities with few side effects. The deficiencies of lower bioavailability of curcumin could be improved by dosage form transformation. However, curcumin in the clinical application still requires further study regarding the underlying mechanism and clinical trial verification.

## 1. Introduction

Curcumin, a diarylheptanoids compound, is a natural active polyphenol extracted from zingiberaceae, including curcumae radix, curcumae longae rhizoma, curcumae rhizoma, and acori tatarinowii rhizoma et al [1]. Curcumin has sufficient and reliable safety with non-toxic properties. Thus, it is one of the most widely used natural edible colorings in the world and is authorized by the United States Food and Drug Administration (FDA) for use as food additives in many countries [2]. In the past decade, the pharmacological activity and mechanism of action of curcumin has attracted more and more researcher attention. With the increasing research, it has been demonstrated that curcumin has the ability of modulating multitarget signal transductions, which plays a significant role in anti-inflammatory [3, 4], antioxygenation, lipid regulation [5], anticancer [6-8], anti-coagulation, anti-atherosclerosis [9] and other pharmacological effects. Particularly in disease prevention, its anti-oxygenation and anti-inflammatory activities have attracted extensive attention from experts all around the world.

Although curcumin has extensive and excellent pharmacological effects mentioned above, there are still obvious problems including poor oral bioavailability and low chemical stability, which limit its clinical application [10-12]. Structural modifications, different delivery systems and curcumin analogues could be conducted to improve curcumin physiochemical properties and increase its bioavailability [13].

**Figure 1.**
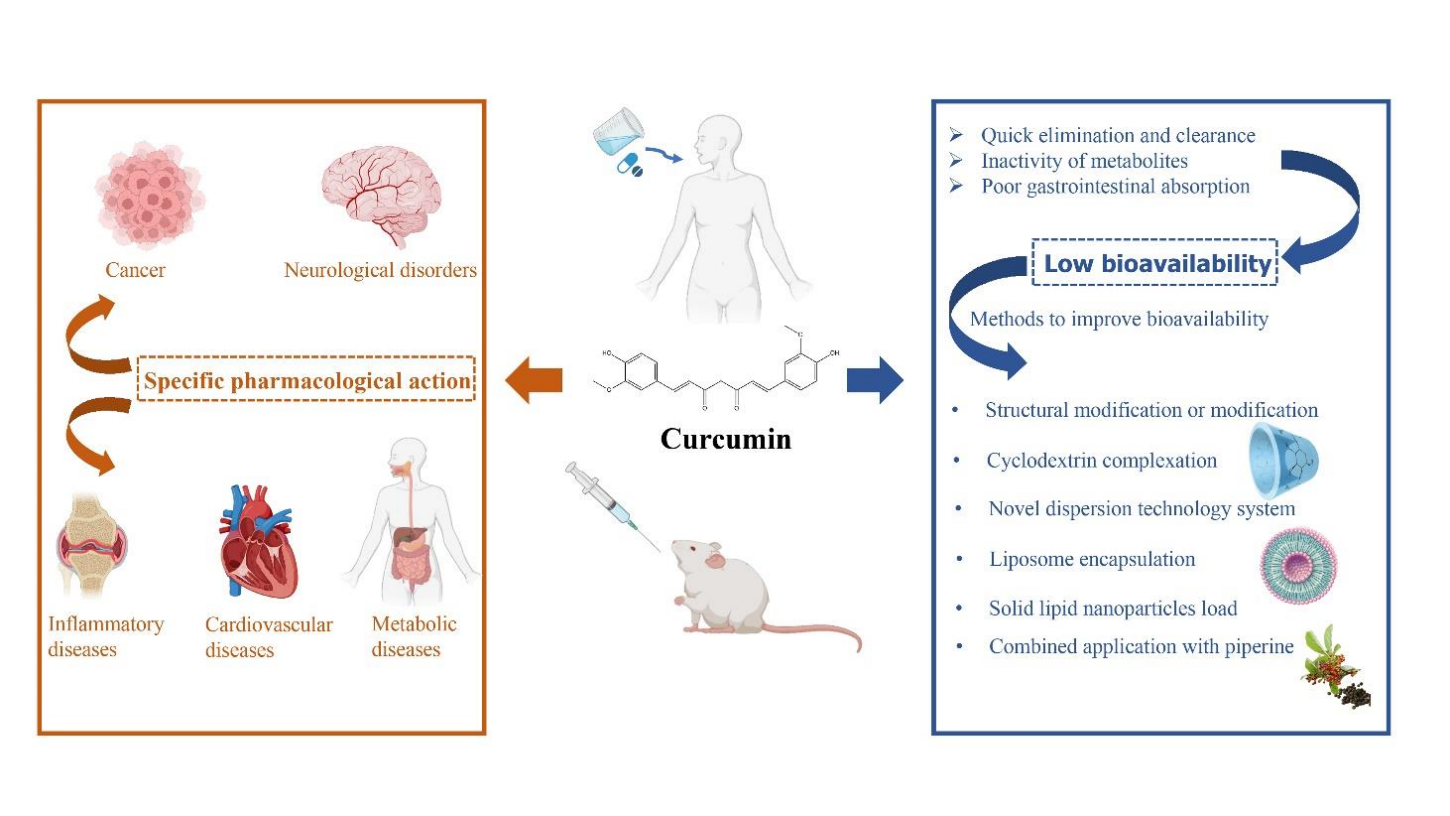
Graphical summary of pharmacological mechanisms and clinical applications of curcumin.

This paper reviews the recent experimental research progress regarding curcumin pharmacological mechanism in recent years. In addition, the obstacles in clinical application and ongoing solutions are summarized (Fig. 1).

## 2. The pharmacological mechanisms of curcumin

**Figure 2.**
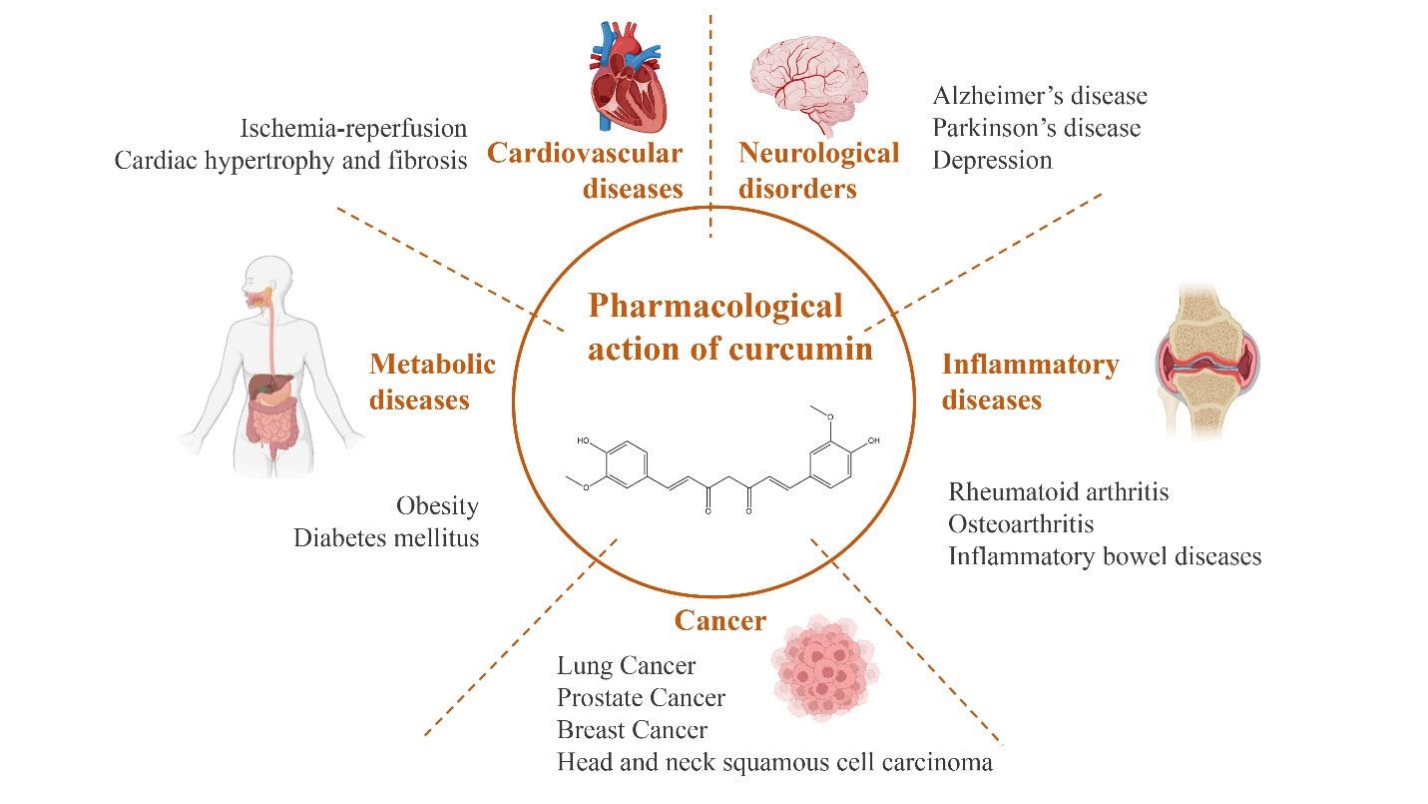
Main pharmacological effects of curcumin.

Traditional and alternative medicine has a significant influence on the trends in preclinical and clinical study of medicinal plants as well as natural active compounds [14-16]. Thus, many literatures show that curcumin has various pharmacologic actions including anti-inflammatory, oxidation resistance, anticoagulant, lipid regulation, and antitumor [3-9]. Currently, studies on its pharmacological mechanisms mainly focus on cardiovascular diseases [17], inflammatory diseases, tumors, and neurological diseases (Fig. 2).

### 2.1 Antitumor effects of curcumin

**Figure 3.**
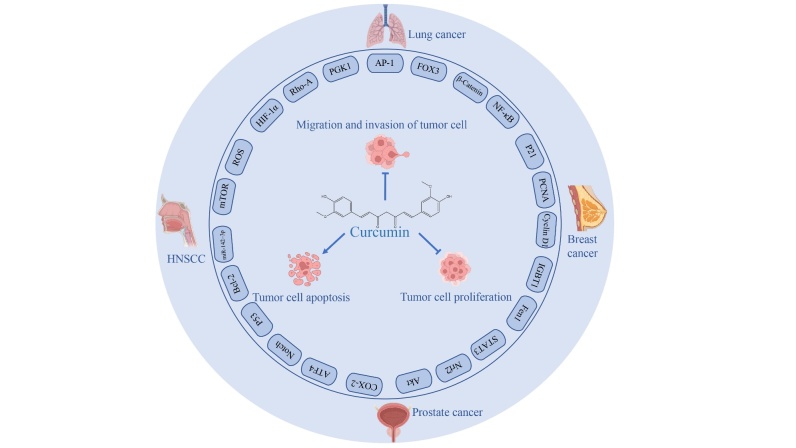
Main molecular mechanism of curcumin on cancer.

Cancer is one of the major public health issues around the world, and the study of anticancer drugs has always been the major task and challenges for medical scientists. The biological characteristics of cancer including abnormal cell differentiation, uncontrolled cell reproduction, invasion and metastasis lead to the cancer formation to be a complex process that involves many factors. The etiology of cancer has not been fully understood, and there is still a lack of ideal and highly specific early diagnosis methods. Therefore, curcumin has gained extensive attention in the past few years because of the anti-tumor activities. Many studies have shown that curcumin has significant effects on various types of cancer including pulmonary tumor, breast carcinoma, prostate carcinoma, head, and neck squamous cell carcinoma. The anticancer mechanisms of curcumin mainly refer to multiple signaling pathways including cell proliferation, apoptosis, migration, invasion, as well as immunoregulation [18, 19]. The specific pharmacological action of curcumin against cancer and its molecular mechanism were summarized in Figure 3 and listed in Table 1.

#### 2.1.1 Anti-lung cancer effect of curcumin

Pulmonary cancer is the primary cause of tumor-related death in the world. It is reported that curcumin could inhibit myeloid-derived suppressor cells (MDSCs) accumulation and reduce the immune suppressive action of MDSCs [20]. In addition, curcumin have shown obvious cytotoxic effect on non-small-cell lung cancer (NSCLC) cells by reducing mitochondrial transmembrane potential and inducing reactive oxygen species (ROS) production through activating the DNA damage/repair pathway and mitochondrial apoptosis mechanism [21]. Other studies also indicated that curcumin could reduce mitochondrial membrane potential and then lead to caspase-9/caspase-3 cascade activation, which eventually give rise to apoptosis in A549 lung adenocarcinoma cells [6]. In addition, calcium overload would cause the mitochondrial-dependent apoptosis of NSCLC cells in the presence of curcumin [22].

In addition, there is evidence to prove that lysosomes are involved in curcumin-induced lung cancer cell apoptosis. Curcumin could trigger a significant lysosomal instability and release lysosomal cathepsin B into the cytosol, which depends on the rise of ROS and is antecedent to the mitochondrial dysfunction [23]. Moreover, curcumin could trigger the autophagy of human lung adenocarcinoma A549 cell and lead to a significant increase of gene expression of human GD3 synthase (hST8Sia I), which could synthesize ganglioside GD3. Under the stimulant of curcumin, activation of nuclear factor Kappa B (NF-κB) induces activation of hST8Sia I gene transcription [24]. Curcumin could reduce the expression of COX-2 through the attenuation of NF-κB binding to decrease survival and increased induction of apoptosis of human NSCLC cell lines [25]. Curcumin also could induce both apoptosis and autophagy in NSCLC A549 cells by inhibiting the PI3K/Akt/mTOR pathway [26]. Through the PI3K/Akt pathway, curcumin could suppress neutrophil elastase-induced tumor cell growth via increasing α1-antitrypsin expression [27]. Many studies have demonstrated that the abnormal miRNAs expression has a significant impact on tumorigenesis, suggesting that the antitumor effect of curcumin is related to miRNAs. Zhan et al through pathway analysis found that mitogen-activated protein kinase (MAPK), transforming growth factor-β (TGF-β), and Wnt signal pathway showed an obvious downward trend. By constructing miRNA gene network that is associated with metastasis-inhibiting metastasis of curcumin in NSCLC A549 cells, miR-330-5p, let-7a-3p, miR-499a-5p, miR-1262, miR-1276, and miR-331-5p were deemed to be pivotal microRNA regulators [28]. By the establishment of a miRNA-transcription factor (TF)-target gene network, Jiao et al found that MiR-34a-5p/ miR-34c-5p/miR-302b-3p—lymphoid enhancer binding factor 1 (LEF1)—cyclin-D1 (CCND1)/Wnt family member 1 (WNT1)/v-myc avian myelocytomatosis viral oncogene homolog (MYC) axis played a role in curcumin-mediated inhibition of lung carcinoma cell metastasis [29]. Among them, the increased miR-192-5p was an important factor [30]. The mechanism of p53 independently inducing apoptosis of lung cancer cells has been proved by investigating the changes at the cellular and molecular level induced under the pharmacologic action of curcumin in A549 (p53 proficient) and H1299 (p53 null mutant) [31]. The p53-miR-192-5p/215-XIAP pathway is considered as an important therapeutic effect target [32]. Curcumin constrained the abnormal proliferation and differentiation of NSCLC cells by inhibiting the circ-PRKCA/miR-384/ITGB1 pathway and adjusting and controlling ITGB1 expression via adsorbing miR-384 [33]. MiR-98, as a tumor suppressor, could be modulated by curcumin to restrain the metastasis of multiple tumor cells. The overexpression of miR-98 suppressed matrix metalloproteinase (MMP)2 and MMP9 through targeting LIN28A, thus inhibiting the abnormal proliferation of human lung tumor cells [34]. Curcumin could inhibit MMP-2 and -9 also through MEKK3, p-ERK signal pathways [35]. In another study, the overexpression of MMP-9 could be restrained by curcumin via the protein kinase Cα (PKCα)/NADPH-Oxidase 2 (Nox-2)/activating transcription factor 2 (ATF-2) signal pathways. PKCα plays a catalytic role in the expression of MMP-9 that rests with Nox-2 expression and ATF-2 phosphorylation [36]. Rac1 is a kind of vital small Rho GTPases family protein and Rac1-regulated actin cytoskeleton rearrangement may play a crucial part in the anti-invasion of curcumin on lung cancer cells. The study also shows that the inhibition of invasion is associated with inhibition of Rac1/PAK1 signal pathway [37]. Cell division control protein 42 homolog (Cdc42), a member of Rho GTPase family, participates in human cancer cell survival, proliferation, transformation, invasion, and metastasis. Literature reported that curcumin could significantly inhibit the overexpression of Cdc42 gene as well as Cdc42-related target gene to regulate the actin cytoskeletal rearrangement and restrict lung cancer cell growth and invasion [38]. Curcumin could suppress the formation of tumor spheres, which has been proved highly associated with neoplasm recurrence and drug fast. Curcumin could inhibit the formation of tumor spheres by restricting the JAK2/STAT3 signal pathway [39] and suppress NSCLC cell migration by reducing the expression of AP-1 protein and suppressing the epithelial-mesenchymal transition (EMT) course, which are mediated by TLR4/MyD88-EGFR pathway [40]. In A549, curcumin could inhibit the Wnt/β-catenin pathway, which is associated with oxidative stress processes [41]. The growth and proliferation of lung cancer stem cells (CSCs) was suppressed by curcumin through Wnt/β-catenin and Sonic Hh pathways [42]. DnaJ-like heat shock protein 40 (HLJ1) is kind of tumor suppressor, and the expression of HLJ1 was proved to be transcriptionally increased by curcumin HLJ1 via an activator protein (AP-1) site in HLJ1 enhancer, particularly, JunD, as a kind of AP-1 components, was obviously increased. Increased HLJ1 expression ulteriorly brings about decrease of E-cadherin, which is a vital component in the compliant connection betwixt epithelial cells and influences inhibiting the invasion of cancer cells [43].

**Table. 1:** Pharmacological mechanism of curcumin on cancers

Research object (Cancer types)	in vitro/ in vivo	administration route	Mechanism of action	Results	Ref.
**Human lung adenocarcinoma A549 cells lines (lung cancer)**	in vitro		↑Bax proteins, cytochrome c, caspase-9, and caspase-3↓ PARP and Bcl-2	Induction of cell death by mitochondria-mediated intrinsic caspase pathways.	[6]
**Breast fibroblast cells (breast cancer)**	in vitro		↑p16INK4A ↓ SDF-1, IL-6, MMP-2, MMP-9, TGF-β and Lamin B1	Induction of possible inactivation of cancer-associated myofibroblasts and trigger DNA damage-independent senescence in stromal fibroblasts.	[8]
**Gr-1^high^Ly6G^+^ (G-MDSCs), Gr-1^dim^Ly-6G^-^ (M-MDSCs) myeloid cells and C57BL/6J mice that were injected subcutaneously with LLC cells (2.5 × 105/mouse, 100 μl in PBS) (lung cancer)**	in vitro in vivo	s.c.	↓immune suppressive factors of MDSCs, argi_x005f_x005f_ x005f_x0002_nase-1 (Arg-1), ROS and IL-6	Induction of the accumulation and function of MDSCs in vitro and vivo.	[20]
**NSCLC cell lines A549 and SPC-A1(lung cancer)**	in vitro		↓ mitochondrial transmembrane potential ↑ induced ROS production	Specific cytotoxicity against NSCLC by activating the DNA damage/repair pathway and mitochondrial apoptosis.	[21]
**A549 and H1299 lung cancer cells**	in vitro		↑[Ca^2+^]i level, ↓Bcl-2,cleaved caspase-3, cleaved caspase-9, ↑phosphorylation level of IP3R	Cytotoxic effects on lung cancer cells were induced by calcium overload.	[22]
**A549 lung cancer cells**	in vitro		↑LMP and the size and number of lysosomes	The participation of lysosomes was proved in lung cancer cell apoptosis.	[23]
**Human lung adenocarcinoma A549 cell line (lung cancer)**	in vitro		↑hST8Sia I and ganglioside GD3	Activation of NF-κB induces transcriptional activation of hST8Sia I gene via AMPK signal pathway.	[24]
**H1975 NSCLC cells, ectopic and orthotopic lung tumor mouse models (lung cancer)**	in vitro in vivo	lung cancer cells in Matrigel were injected percutaneously into the left lung of CD-1 nude mice.	↓IkB, nuclear p65, COX-2 and p-ERK1/2	The tumor growth of orthotopic human NSCLC xenografts and the survival of treated athymic mice were significantly reduced.	[19]
**NSCLC A549 cells (lung cancer)**	in vitro		↑Beclin1 and LC3-II expression, ↓p62 protein, mTOR and Akt	Induction of both apoptosis and autophagy via PI3K/Akt/mTOR pathway.	[26]
**NSCLC A549 cells and C57BL/6 mice (lung cancer)**	in vitro in vivo	s.c.	↑α1-antitrypsin, ↓neutrophil elastase	The inhibition of neutrophil elastase-induced proliferation by increasing α1-antitrypsin in vitro and in vivo.	[27]
**NSCLC A549 cells (lung cancer)**	in vitro		↓mitogen-activated protein kinase, transforming growth factor-β, and Wnt signaling pathways and ↑axon guidance, glioma, and ErbB tyrosine kinase receptor signaling pathways	Construction of a miRNA gene network that conduced to restricting metastasis in lung cancer cells.	[28]
**Human high-metastatic non-small cell lung cancer 95D cells**	in vitro		The construction of a miRNA-transcription factor (TF)-target gene network to clarifying the suppression mechanisms for lung cancer metastasis.	MiR-34a-5p/miR-34c-5p/miR-302b-3p —LEF1—CCND1/WNT1/MYC axis may be a key mechanism in inhibition of lung cancer metastasis.	[29]
**Human normal NCL-H460 and BEAS-2E lung epithelial cells, and human A549 lung cancer cells**	in vitro		↑caspase-3 activity, ↓miR-192-5p, ↓PI3K/Akt protein	Inhibition of cell proliferation and the induction of apoptosis by upregulating miR192-5p and inhibiting PI3K/Akt signaling pathway.	[30]
**Human lung cancer cell lines-A549 and H1299**	in vitro		↓p53, bcl-2, and bcl-X(L), ↓Bak and Caspase genes	Enhancement of apoptosis and inhibition of cell cycle in lung cancer cells.	[31]
**Human NSCLC H460, A427, A549 and H1299 cells**	in vitro		↓oncogenic miR-186 expression, ↑miR-215 and miR-194-5p, ↓miR-223-3p and miR-60	Proapoptotic impacts of curcumin was associated with miR-192-5p/215 induction.	[32]
**Nude mice were subcutaneously injected with A549 cells carrying sh-circ-PRKCA; NSCLC cells (H1650, H1299, H460, and A549), human bronchial epithelial cell line (16HBE), and 293T cells (lung cancer)**	in vitro in vivo	p.o.	↓circ-PRKCA↑miR-384↓ITGB1	Inhibit the malignancy of NSCLC cells; inhibited xenograft tumor growth through circ-PRKCA	[33]
**Male SCID mice subcutaneously inoculated (in the flanks) with A549 cells (lung cancer)**	in vitro in vivo	i.p.	↓MMP 2, MMP9 and miR-98 ↓LIN28A	Inhibition the growth of human lung cancer cells in vitro and in vivo.	[34]
**NSCLC A549 cells (lung cancer)**	in vitro		↓VEGF, c-jun-p, GRB2, MEKK3, FAK, MKK7, MMP-2, MMP-9, and Rho A ↑JNK and PERK	Inhibition of migration and invasion in A549 cells.	[35]
**NSCLC A549 cells (lung cancer)**	in vitro		↓PKCα, Nox-2, MMP-9 and phosphorylation of ATF-2 ↓ROS	Inhibition of lung cancer A549 cells invasiveness.	[36]
**Human large cell lung carcinoma 801D cell line (lung cancer)**	in vitro		↓Rac1 protein, MMP-2, MMP-9 and PAK1 phosphorylation	Inhibition of migration and invasion of lung cancer cells via Rac1/PAK1 signaling pathway.	[37]
**Human lung cancer cell lines (95D, 801D, A549, and 95C) and human bronchial epithelial cells (BEAS-2B)**	in vitro		↓Cdc42 gene and Cdc42-related target gene	Suppression of invasion that was mediated by Cdc42 in lung cancer cells.	[38]
**Lung cancer cell line NCI-H460 and lung cancer xenograft nude mouse model (lung cancer)**	in vitro in vivo	i.p.	↓p-JAK2 and p-STAT3↓cyclin D1 and C-myc	Inhibition of the formation of tumor spheres.	[39]
**The NSCLC specimens; NSCLC cell lines NCI-A549 and NCI-H226 (human lung squamous cancer)**	in vitro		↓ cyclins ↓CDKs modulated ↓c-Jun and c-Fos ↑E-cadherin ↓vimentin ↓TLR4/MyD88 and EGFR	Block of NSCLC proliferation and metastasis.	[40]
**NSCLC A549 cell (lung cancer)**	in vitro		↓ROS, β-catenin, p-GSK3β, cyclin D1 and c-Myc ↑ SOD and γ-GCS	Inhibition of NSCLC proliferation through the Wnt/β-Catenin pathway which was mediated by oxidative stress.	[41]
**The lung cancer cell line A549 and H1299**	in vitro		↓PCNA, Cyclin D1 and Bcl2↑ Bax and caspases (Caspase 8, Caspase 9, Caspase 3) ↓ p-GSK3β (Ser9) ↑ GSK3β↓β-catenin and its downstream targets (c-Myc and Cyclin D1) ↓Sonic Hh pathway components, including shh, Smo, Gli1 and Gli2	Inhibition of lung CSCs through the Wnt/β-catenin and Sonic Hh pathways.	[42]
**mice with scramble or HLJ1 siRNA-treated CL1-5 cells; The human lung adenocarcinoma cell lines with less invasive (CL1-0) and highly invasive capacities (CL1-5) (lung cancer)**	in vitro in vivo	p.o.	↑JunD, c-Jun NH2-kinase phosphorylation, HLJ1 and E-cadherin	Inhibition of cancer cell migration, invasion, and metastasis.	[43]
**DU145 and PC-3 PC cell lines (prostate cancer)**	in vitro		↓EGFR expression levels and ERK activation ↓the size of DU145 and PC-3 spheroids	Suppression of the size and the viability of PC-derived spheroids via curcumin alone or combinatorial means.	[44]
**The androgen-sensitive human prostatic carcinoma cell line LNCaP (prostate cancer)**	in vitro		↓bcl-2, bcl-XL and androgen receptor	Down-regulation of the androgen receptor protein was the key to therapeutic approaches of AR-dependent prostate cancer.	[45]
**The human prostate cancer cell line PC-3 and CD-1 Foxn1nu male mice(prostate cancer)**	in vitro in vivo	p.o.	↓CXCL1 and -2 ↓the phosphorylation of IκBα, mRNA expression of the metastasis-related genes SPARC, COX2, ALDH3A1 and EFEMP	Suppression of the metastasis prone phenotype formation in prostate cancer cells by inhibiting NFκB signaling.	[46]
**Human androgen-independent (DU145) and -dependent (LNCaP) prostate cancer cell lines(prostate cancer)**	in vitro		↓Bcl-2 and Bcl-xL ↑procaspase-3 and procaspase-8	Survival mechanisms of prostate cancer cells were restricted by inhibiting NF-kappaB and AP-1.	[47]
**Prostate cancer-associated fibroblasts, PC-3 (a prostate cancer cell line), and NAFs cells (prostatic cancer)**	in vitro		↑ROS, the phosphorylation of PERK, eIF2α, CHOP and ATF4	Up-regulation ROS by triggering endoplasmic reticulum stress of CAFs through the PERK-eIF2α-ATF4 axis to inhibit prostate-CAFs apoptosis and cell cycle arrest.	[48]
PC3 cells and human prostate CAFs (prostatic cancer)	in vitro		↓CXCR4, IL-6, ROS, and vimentin ↑E-caderin	Protective effect in EMT process, which is related to the ability of reducing CAF-induced ROS production via MAOA/mTOR/HIF-1α signal pathway.	[49]
**Castration-resistant prostate cancer (prostatic cancer)**	in vitro		↑TfR1and IRP1	Induction of apoptosis and protective autophagy that partially relied on iron-chelating properties.	[50]
**Human prostate cancer cell lines LNCaP and 22Rv1, C57BL/6 mice and heterozygous female TRAMP mice (prostatic cancer)**	in vitro in vivo	p.o.	↓steroidogenic acute regulatory proteins, CYP11A, 1HSD3B2 and testosterone levels ↑AKR1C2	Inhibition of androgen production may be potent anticancer properties and therapeutic effects on prostate cancer.	[51]
**LNCaP cells (prostatic cancer)**	in vitro		↓AR, β-catenin, phosphorylation of Akt and glycogen synthase kinase-3b, cyclin D1 and c-myc, the target gene of the b-catenin/T-cell factor transcriptional complex ↑phosphorylated β-catenin	Inhibitory impacts on LNCaP prostate cancer cells via Wnt/β-catenin signaling pathway.	[52]
**LNCaP cells (prostatic cancer)**	in vitro		↓ NKX3.1 and NKX3.1 1040 bp promoter ↓the binding activity to ARE	Down-regulation of NKX3.1 expression that is deemed to have an key effect on normal prostate organogenesis and carcinogenesis.	[53]
**Human prostate cancer 22RV1, PC-3, and DU145 cells (prostatic cancer)**	in vitro		↑miR-34a and p21↓β-catenin, c-myc, cyclin D1 and PCNA	Inhibition of proliferation of prostatic cancer cells.	[54]
**DU145 prostate cancer cells**	in vitro		↑miR-143 and FOXD3↓ Phosphoglycerate Kinase-1 (PGK1)	Inhibition of prostate cancer.	[55]
**DU145 prostate cancer cells**	in vitro		↓Cyclin D1, CDK2 and Bcl-2 ↑p21, p27, p53 Caspase-3 and Caspase-9	Induction apoptosis and G0/G1 arrest via Notch signaling.	[56]
**DU145 and PC3 cells (prostatic cancer)**	in vitro		↓MT1-MMP, MMP2 and DNA-binding ability of NICD	Inhibition of the survival and metastasis of prostate cancer cells by Notch-1 signaling pathway.	[57]
**LNCaP cells (prostatic cancer)**	in vitro		↑HDAC1, 4, 5, and 8 ↓HDAC3 and enrichment of H3K27me3	Potential epigenetic modifying effect that relied on the CpG demethylation ability.	[58]
**C4-2B prostate cancer cells**	in vitro		↓core-binding factor a-1 and IKK	Suppression of the bony metastases establishment by inhibiting the growth factor collaboration between the prostate cancer cells and the osteoblast/stromal cells.	[59]
**Human triple negative breast cancer cell line (breast cancer)**	in vitro		↑intracellular iron ↑ROS, lipid peroxides, and malondialdehyde ↓glutathione levels ↑HO-1	Triggering action of the molecular and cytological characteristics of ferroptosis.	[63]
**MCF-10F, a normal cell line used as a control; Alpha5, a pre-malignant and tumorigenic cell line; and Tumor2, derived from Alpha5 after the injection of that cell line into nude mice (breast cancer)**	in vitro		↑the proportion of CD44+/ CD24+cells ↓ the proportion of CD44+/CD24-cells	Effect on reducing cancerous types of breast cells by regulating the expression of the cell surface markers CD44 and CD24.	[64]
**Human metastatic breast cancer cells MDA-MB-231**			↓CXCL1 and -2 ↑miR181b	Inhibition of metastatic potential by impacting miRNA expression and pro-inflammatory cytokine CXCL1 in primary tumors.	[65]
**Female BALB/c nude mice subcutaneously inoculated into the right-side flank area with 6 MDA-MB-231 cells; Triple-negative breast cancer (TNBC) MDA-MB-231 cells (breast cancer)**	in vitro in vivo	p.o.	↓ PSMB5 protein and chymotrypsin-like (CT-l) activity of proteasome 20S core↑miR-142-3p p300	Inhibited cell proliferation and cancer growth through p300/miR142-3p/PSMB5 axis	[66]
**MCF-10F human breast and MDA-MB-231 human breast cancer cell lines (breast cancer)**	in vitro		↓Axl, Slug, CD24 and Rho-A gene transcript levels	Effects on expression of related genes in EMT and invasion by regulating miR-34a expression in the breast cell lines.	[67]
**Breast cancer stem cells**	in vitro		↓beta-catenin nuclear translocation, trans-activation of Slug ↑E-cadherin	Inhibition of bCSC migration by amplifying E-cadherin/ β-catenin negative feedback loop.	[68]
**Breast cancer cell lines (MDA-MB-231 and BT-483)**	in vitro		↓cyclin D1, CDK4 and MMP1	Inhibition of breast cancer cell proliferative rate and invasion by suppressing NF-κB inducing genes.	[69]
**ER-negative human breast cancer cell line resistant to chemotherapy, MDA.MB231 and mouse model of breast cancer**	in vitro in vivo	s.c.	↓NF-κB, PECAM-1, cyclin D1, and p65	Inhibition of tumor growth and angiogenesis in mouse model of human breast cancer.	[70]
**MCF-7 (HTB 22) cells (breast cancer)**	in vitro		↓cyclin B1, Cdc2, CDK4, CDK2, cyclin E2 and the nuclear transport of NFκB	Induction of cell cycle arrest by regulating of NF-κB and polyamine biosynthesis.	[71]
**MDA-MB-231, MDA-MB-468, and MCF-7 breast cancer cells**	in vitro		↓the mRNA and protein levels of visfatin, NF-κB and p65	Down-regulation of visfatin gene expression in human breast cancer cells via NF-κB dependent.	[72]
**Human breast cancer MDA-MB-231 cells**	in vitro		↓FAS activity and Bcl-2 ↑the phosphorylation of Akt and Bax	Induction of cell apoptosis by inhibiting FAS.	[73]
**MDA-MB-231 breast cancer cells**	in vitro		↓PTGS2/COX2, CXCL1, CXCL2 and EGR1 ↑GCLM and HMOX1	Inhibition of breast cancer metastasis via the suppression of NFjB activation and the expression of two prometastatic cytokines, CXCL1 and -2.	[74]
**Paclitaxel (Taxol)-resistant breast cancer cells and a human breast cancer xenograft model (breast cancer)**	in vitro in vivo	p.o.	↓antiapoptotic (XIAP, IAP-1, IAP-2, Bcl-2, and Bcl-xL) ↓ proliferative (cyclooxygenase 2, c-Myc, and cyclin D1), ↓metastatic proteins (vascular endothelial growth factor, matrix metalloproteinase-9, and intercellular adhesion molecule-1)	Inhibition of breast cancer metastasis via suppression of NF-κB and NF-κB-regulated gene products.	[75]
**Human breast cancer cell line MCF-7**	in vitro		↓Fen1↑Nrf2	Inhibition the proliferation of breast cancer cells by Nrf2-mediated down-regulation of Fen1 expression.	[76]
**Antiestrogen-resistant breast cancer cell lines MCF-7/LCC2 and MCF-7/LCC9**	in vitro		↓EZH2, p65, IκBα, cyclin D1 and c-Myc, Bcl-2 and Bcl-xL	Therapeutic benefit for endocrine-resistant breast cancer.	[77]
**TNBC MDA-MB-231 cells and ERα) positive breast cancer MCF7 cells (breast cancer)**	in vitro		↓the phosphorylation of mTOR, SLUG and HK2 ↑Cyt C and the cleavage of caspase 3	Overcoming 4-OHT resistance of TNBC by targeting SLUG.	[78]
**Human breast cancer cells (MCF-7 cells)**	in vitro		↓SERPINE1 ↑PGAP3, MAP3K1, SERPINE1, PON2, and GSTO2	Potential therapeutic in breast cancer by regulating breast cancer-related genes, including SERPINE1, PGAP3, MAP3K1, MAPK1, GSTO2, VIM, SPARC, and FGF2.	[79]
**Female athymic nude mice subcutaneously injected with HNSCC cell line in the left or right flank; HNSCC cell lines CCL 23, CAL 27, and UM-SCC1 (HNSCC)**	in vitro in vivo	intratumoral injection	↓p16, cyclin D1, phospho-Iκβ, and NF-κB	Inhibition of HNSCC multiplication in vitro and in vivo	[81]
**UDSCC1 and UDSCC4 cell lines (HNSCC)**	in vitro		↓NF-κB↓Treg attraction↓key transcription factors of EMT, Snail, and Twist↓Treg-attracting chemokine CCL22	Reversal the EMT of HNSCC cells and the immunomodulatory effects.	[82]
**Salivary cells of HNSCC patients; UM-SCC1 cells (HNSCC)**	in vitro in vivo	p.o.	↓proinflammatory cytokines(IL-10, IFN-γ, IL-12p70, IL-2, granulocyte macrophage colony stimulating factor and TNF-α)↓ IKKb ↓NF-κB	Inhibition of IKKβ kinase activity in the salivary cells of HNSCC patients	[83]
**MDA 686LN cells (HNSCC)**	in vitro		↓Bcl-2, cyclin D1, COX-2 and MMP-9↑upstream and downstream caspase↑	Suppression of cell cycle at G1/S stage and induce cell death in MDA 686LN cells.	[84]
**Female athymic nude (nu/nu) mice transplanted with FaDu cells;HNSCC cell lines including FaDu and Cal27 cells (HNSCC)**	in vitro in vivo	p.o.	SIRT1 signal pathway ↓MMP-2 and VEGF protein ↑ATM-CHK2/caspase 8/9 pathway	Potent anticancer activity via SIRT1 pathway both in vitro and in vivo.	[85]
**Human HNSCC cell lines MDA 1986, Tu 686, Tu 167, MDA 686LN and JMAR (HNSCC)**	in vitro		↓STAT3 phosphorylation ↓Nuclear translocation of STAT3↓IL-6-induced activation of STAT3	Suppression of the proliferation of HNSCC cells.	[86]
**The Balb/c nude mice inoculated subcutaneously with curcumin treated HuPCaSCs; CD44^+^/CD133^+^ human prostate cancer stem cells (HuPCaSCs) isolated from the prostate cancer cell lines Du145 and 22RV1 (prostatic cancer)**	in vitro in vivo	inoculated subcutaneously with curcumin treated HuPCaSCs	↓cell cycle proteins (Ccnd1 and Cdk4) and stem cell markers (Oct4, CD44, and CD133) ↑miR-145↓lncRNA-ROR	Inhibited CD44+/CD133+ HuPCaSCs xenograft growth; Inhibition of the growth, invasion, and tumorigenicity of HuPCaSCs.	[223]
**Human breast cancer cell line MDA-MB-361 (breast cancer)**	in vitro		↑DLC1 ↓Sp1 ↓DNA methyltransferase 1 ↓RhoA and Cdc42	Inhibition of the expression of Sp1 to down-regulate DNA methyltransferase 1.	[224]

#### 2.1.2 Anti-prostate cancer effect of curcumin

Prostate cancer is a common internal malignant tumor in men as well as the second leading cause of male cancer-related deaths in the world [44]. Therefore, it’s very important to find effective means for the prompt diagnosis and therapy of prostate cancer. Evidence indicates that curcumin can efficiently restrain the cell multiplication, invasion as well as tumorigenesis of prostate cancer cells in vitro and in vivo [38, 39, 45]. Chronic inflammation can promote cell metastasis in prostate cancer cells by sustaining a positive feedback loop between NF-κB and chemokine (C-X-C motif) ligand 1/2 (CXCL1/2). Curcumin blocks this feedback loop by inhibiting NF-κB signaling pathway leading to reduced metastasis formation in vivo [46]. Curcumin would cause apoptosis in both androgen-dependent LNCaP and androgen-independent DU145 cell lines though down-regulated Bcl-2 and Bcl-xL expression as well as the excitation of procaspase-3 and procaspase-8 [41]. Protein kinase B (PKB/Akt) is a serine-threonine protein kinase and is related to cell multiplication, cell cycle progression and apoptosis. By inhibiting the activation of Akt, curcumin could induce programmed cell death in human prostate cancer cell lines, LNCaP and PC-3 [7]. Literature reported that cancer-associated fibroblasts (CAFs) have an important influence on tumorigenesis, development, and migration. Curcumin could inhibit prostate-CAFs apoptosis and arrest cell cycle at G2-M phase via ROS-mediated endoplasmic reticulum stress pathway through the PERK-eIF2α-ATF4 axis [48]. CAFs also could affect epithelial to EMT and induce cencer stem cell properties. Curcumin blocks prostate carcinoma cell invasion and EMT that induced by CAFs and reduce the generation of ROS by inhibiting the monoamine oxidase A (MAOA) /mammalian target of rapamycin (mTOR)/hypoxia-inducible factor-1α (HIF-1α) signaling [49]. Curcumin is an active iron chelator with biological activity and thus produces cytotoxic effects. In castration-resistant prostate cancer (CRPC), curcumin could induce the apoptosis and protective autophagy, which depended at least in part on its iron-chelating properties [50]. Intratumoral androgen biosynthesis has been deemed an important element of castration-resistant prostate cancer. Curcumin may play an important role in inhibiting carcinogenesis and development by facilitating the reduction of intracellular prostate testosterone and inhibiting testosterone activity in LNCaP. Therefore, curcumin regulates the intracellular activity of androgen signaling directly and indirectly [51, 52]. NKX3.1 has been proved to be an androgen-regulated NK-class homeobox gene that is the most likely specific expression in the prostate, and greatly affects organogenesis and carcinogenesis of normal prostate. Curcumin inhibits the androgen receptor (AR)-mediated expression of NKX3.1 by decreasing AR expression and blocking its DNA binding activity in LNCaP cells [53]. MicroRNA-34a (miR-34a) is a major tumor inhibitor and, regulates the expression of β-catenin and c-myc. The latter is an oncogene that activates cell cycle-boosting genes and inhibits cell cycle-suppressing genes. Curcumin stimulates the expression of miR-34a, on the contrary down-regulate the expression of β-catenin and c-myc, and also directly influenced the activation of cell cycle regulation genes (cyclin D1, PCNA, and p21) [54]. In DU145 prostate cancer cells, curcumin was found to upregulate Forkhead D3 (FOXD3), a transcriptional factor of miR-143, and thus miR-143 expression could be increased. Therefore, Phosphoglycerate Kinase-1 (PGK1), a biomarker related to the aggressiveness of prostate tumor cells, would be downregulated by miR-143 [55]. The abnormal activation of Notch appeared in many malignancies, including malignant prostates, and Notch dysfunctional would guide the transformation of the undifferentiated cells into malignancies by inhibiting cell differentiation. Curcumin downregulates Notch signal pathway to promote apoptosis as well as arrest cell cycle at G0/G1 [56, 57]. Neurog1, a basic helix-loop-helix protein, would affect neuronal differentiation and more importantly, the highly methylated Neurog1 would be disturbed in prostate cancer and thus be chosen as one of the cancer methylation markers. In the study of human LNCaP prostate cancer cells, curcumin could demethylate the Neurog1 promoter and reactivate its mRNA and protein expression, which indicated that curcumin may be promising in epigenetic therapy of prostate cancers [58]. Curcumin may inhibit the formation of bony metastases through the ability of disturbing osteoblastic component and osteoclastic component of osteomimetic properities via the growth factor receptor pathways and NF-κB activation process in C4-2B, a prone metastatic derivative in LNCaP [59].

#### 2.1.3 Anti-breast cancer effect of curcumin

Breast cancer, the most frequent invasive cancer in female, is also the 2^nd^ cause of cancer-related death among women [60]. Many studies have been performed to explore the active mechanism of curcumin on breast cancer.

High glucose consumption is a basic metabolic characteristic of cancer tissues [61]. Literature reported that curcumin could improve triple negative breast cancer by regulate key glycolysis proteins [62]. Activated cancer-associated fibroblasts promote tumor growth and spread as well as influence tumor response to therapeutic drugs. Curcumin induces senescence in activated cancer-associated fibroblasts, which causes these cells to death and reduce their procarcinogenic potential [8]. Curcumin significantly restricts the survival of breast cancer cells *via* upregulating many ferroptosis target genes involved in redox regulation, in especial heme oxygenase-1 (HO-1) [63]. In human mammary cell lines, the cell surface markers CD44 belong to type I transmembrane glycoprotein, which could adjust cell adhesion, cell to cell and cell to extracellular matrix mutual effects. CD24 is usually found in benign and malignant solid tumors and associated with cell adhesion and metastasis as well. Curcumin could decrease cancerous types of breast cells by improving the proportion of CD44^+^/CD24^+^ cells [64]. In terms of suppressing the invasion and migration of metastatic breast cancer cells, curcumin has been proved to induce the activation of many miRNAs, such as miR181b. Curcumin also down-regulated the pro-inflammatory cytokine CXCL1 and -2 in cells separated from some essential human breast cancers, which are possible targets of miR181b [65]. Curcumin, as a proteasome inhibitor, could inhibit p300/miR-142-3p/PSMB5 axis associated with the restriction of the CT-l viability of 20S proteasome in triple-negative breast cancer (TNBC) MDA-MB-231 cells [66]. Curcumin could regulate miRNAs by controlling relative genes involved in EMT, as well as Ras homolog gene family member A (Rho-A), impressing the migration and invasion of the non-malignant MCF-10F and malignant MDA-MB-231 breast cancer cell lines [67]. Breast cancer stem-like cells (BCSCs) have remarkable migration characteristics because of the inherent restriction of the tumor inhibiting factor, E-cadherin, which could be recovered by curcumin. Curcumin inhibits β-catenin nuclear translocation and ultimately inhibit EMT and the migration of BCSCs [59]. In both ER-positive BT-483 and ER-negative MDA-MB-231 breast cancer cell line, curcumin showed prominent impact on mediating the proliferative ratio and invasion through the downregulation of NF-κB induced genes [69]. In a heterotopic mouse model of breast cancer, curcumin suppressed tumor cells growth and angiogenesis through deregulating the activation of NF-κB -adjusted gene products (cyclin D1, PECAM-1, and p65) [70]. Curcumin blocks cell cycle at G2/M phase and lead to cell death via up-regulating SSAT, an enzyme that is regulated by NF-κB. The rate of ROS generation was also increased and then lead to trigger apoptosis by inducing DNA damage [71]. In MDA-MB-231, MDA-MB-468, and MCF-7 breast cancer cells, curcumin could reduce mRNA and protein expression of visfatin, and decrease the activity of constitutive nuclear factor NF-κB. The study also identified two assumed NF-κB-binding sites at -484 and -430 bp relative to the transcription start site on visfatin promoter [72]. High expression of fatty acid synthase (FAS) was discovered in many cancer cells, involving breast cancer, meanwhile it was also demonstrated that curcumin could accelerate cell death by suppressing the expression of FAS [73]. Curcumin inhibits breast cancer cell metastasis by reducing the expression of chemotactic cytokines CXCL1 and -2 whose messenger RNA level rely on NF-κB and needs complete IκBα expression [74]. Curcumin could strengthen the impact of chemotherapy on terminal breast cancer and restrain metastasis by suppressing the paclitaxel-induced NF-κB pathway. Moreover, the effect of curcumin was mediated via the inhibition of IκBα kinase activation as well as IκBα phosphorylation and degradation [75]. Flap endonuclease 1 (Fen1) is a particular nuclease involved in DNA restoration and NF-E2-related factor 2 (Nrf2), as a transcription factor, has a staple regulatory effect on cellular antioxidant defense systems. Curcumin is capable of suppressing the proliferation of breast cancer cells via Nrf2-mediated reduction of Fen1 activation [76]. In the antiestrogen-resistant MCF-7/LCC2 and MCF-7/LCC9, curcumin induced cell death and recovered Tamoxifen sensibility by inhibiting the activation of NF-κB, Src and Akt/mTOR pathways and downregulate the key epigenetic modifier EZH2 proteins [77]. Curcumin could promote 4-hydroxytamoxifen sensitivity in MDA-MB-231 cell line via suppressing SLUG/Hexokinase 2 pathway. In terms of mechanism, SLUG activates HK2 transcription through the combination with its promoter [78]. Besides, curcumin might play a potential therapeutic role in breast tumor by controlling breast tumor-relevant genes including SERPINE1, PGAP3, MAP3K1, MAPK1, GSTO2, VIM, SPARC, and FGF2, which need to validate in the future [79]. In vivo and in vitro studies indicated that curcumin could mediate inhibition of androgen and estrogen signaling pathways to exert its antitumor effect in the reproductive system in females and males [80].

#### 2.1.4 Anti-head and neck squamous cell carcinoma effect of curcumin

Head and neck squamous cell carcinoma (HNSCC) are the sixth most familiar type of cancer around the world and has been proved to inhibit inherent anticancer immunity and to reduce signals of antitumor immune response, which eventually lead to tumor growth and progression.

Curcumin treatment could inhibit HNSCC cell growth in vitro and in vivo by inhibiting the protein expression of p16, cyclin D1, phospho-Iκβ, and NF-κB [81]. Curcumin has the potential of reversing the EMT of HNSCC cells and the immunomodulatory effects that could suppress the regulatory T-cell (Treg)-attracting impacts of toll-like receptor 3 (TLR3) agonist Poly I:C (PIC) [82]. IκBkinase (IKK) kinases including IKKα, IKKβ, IKKγ are regarded as the NF-κB essential modulator. Evidence has shown that curcumin could bind to the IKK kinases to exert the effect of inhibiting NF-κB transcription activation, which eventually reduced cell proliferation of salivary cells in HNSCC patients. Also, the inhibiting impact of curcumin was associated with the down-regulated expression of certain cytokines, including IL-10, IFN-γ, IL-12p70, IL-2, granulocyte macrophage colony stimulating factor and TNF-α [83]. Results of data showed that the expression of some cell living and cell growth genes covering Bcl-2, cyclin D1, COX-2 and MMP-9. In MDA 686LN cells, curcumin could arrest cell cycle at G1/S stage and induce cell death by activating upstream and downstream caspase, PARP cleavage, annexin V staining [84]. SIRT1 is a mammalian homolog of Sir2 that is involved in modifying both histone and non-histone proteins and plays a role in apoptosis, cell proliferation and metabolism. Besides, SIRT1 also regulates p53, NF-κB and Fork head transcription factors and induces apoptosis by activating the ATM-CHK2/ caspase-8/9 pathway in FaDu and Cal27 cells. Using Xenograft mouse model, studies have also demonstrated that the anticancer activity of curcumin in vivo depends on SIRT1 pathway [85]. Many studies indicate that interleukin-6 (IL-6) accelerates cancer cells survival and proliferation through the phosphorylation of a cell-signaling protein, STAT3. Chakravarti et al found that curcumin could suppress STAT3 phosphorylation and nuclear translocation, which was induced by IL-6, and ultimately inhibit the proliferation of HNSCC cells [86].

### 2.2 Therapeutical effect of curcumin on neurological disorders

**Figure 4.**
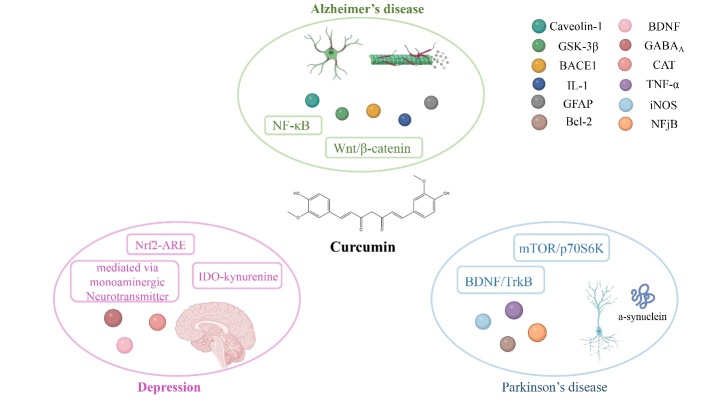
Main molecular mechanism of curcumin on neurological disorders.

Neurological diseases include diseases of the brain, spine, and nervous system. Current studies have shown that curcumin has therapeutic effects on Alzheimer's disease (AD), Parkinson’s disease (PD), and depression. The pharmacological action of curcumin against neurological disorders and its molecular mechanism are shown in Figure 4.

#### 2.2.3 Therapeutical effect of curcumin on AD

AD is the most common cause of dementia, which is an increasing global health issue with huge influences for humans. Curcumin could induce neurogenesis by targeting endogenous neural stem cells (NSC) to influence the brain self-regenerative capacity. Curcumin encapsulated PLGA nanoparticles (Cur-PLGA-NPs) increases NSC proliferation and neuronal differentiation in the hippocampus and subventricular zone of adult rats by Wnt/β-catenin pathway. These findings may provide a way to enhance the brain self-repair mechanism for fighting against neurodegenerative diseases such as AD [77].

Inhibiting the pathological accumulation of tau is regarded as an approach for the treatment of many neuronal diseases including AD and PD. This study found a feasible binding site of curcumin in the microtubule-binding region of tau by Molecular docking studies by inhibiting the aggregation and the oligomerization of tau. The underlying mechanism may be to inhibit the-sheets formation and tau fibril formation in tau [88]. Tau is abnormally phosphorylated in AD brains. A study proved that curcumin could restrain abnormal hyper-phosphorylation of Tau by inactivating the Caveolin-1/GSK-3β pathway. Caveolin-1, the marker protein of membranal caveolae, is closely related to Tau. GSK-3β was the key protein kinase of Tau among the kinases and phosphatases in the Tau phosphorylation. Experimental data show that curcumin could decrease the number of caveolae, made the membrane thinner and could decrease the expression of Caveolin-1, but increase the expression of phosphorylated GSK-3β [89].

As one of the rate-limiting proteases in amyloid-β (Aβ) generation, β-site amyloid precursor protein-cleaving enzyme 1 (BACE1) inhibition is also a target for potential treatment of AD. The study found that curcumin could notably reduce Aβ levels in HEK293-APPswe cells and inhibit BACE1gene expression in SH-SY5Y cells. Further research found that both the inhibition of BACE1 and activation of estrogen receptor beta (ERβ) by curcumin is related to the NF-κB signal pathway [90].

Studies demonstrated that after the treatment of curcumin, senile plaques were marked to clear and reduce existing plaques in APPswe/PS1dE9 mice. Plaque deposition is closely related to neurotoxicity, which exhibited dystrophies and distorted neurites. Curcumin was also proved to have a degree of reversal of structural changes in dystrophic dendrites [91]. In AD genetics, the Aβ phagocytosis of microglial can be activated by the innate immune genes, TREM2 (Triggering Receptor Expressed on Myeloid cells 2) and CD33, products that oppose each other in the downstream Syk tyrosine kinase pathway. TREM2 and CD33 belong to the network of AD-dysregulated innate immune genes controlled by hub gene TyroBP (DAP12), which controls phagocytosis markers (CD68 and Arg1)as well. Previous studies also showed that curcumin decreased CD33 and increased TREM2 and TyroBP and reduced the levels of miR-155, a micro-RNA that can drive a neurodegenerative microglial phenotype. Taken together, Curcumin could stimulate phagocytic clearance of amyloid as an immunomodulatory treatment by restoring neuro-inflammatory networks in neurodegenerative diseases [92].

Recent studies showed that astrocytes (AS) are key factors in the early pathophysiological changes of AD, and glial fibrillary acidic protein (GFAP) is a specific marker of AS. The study investigated the influence of curcumin on amyloid-β (Aβ_1-40_) induced AD rat models at behavioral and molecular levels and found that curcumin significantly improved spatial memory ability, GFAP mRNA expression as well as total GFAP positive cells in AD rats [93].

Increased cytokines and microglial activation are inflammatory manifestations in patients with AD. Curcumin, as a non-steroidal anti-inflammatory drug, reduces oxidative damage, inflammatory cytokine IL-1 and astrocyte inflammatory marker GFAP, thereby reducing the risk of inflammation [84]. AD might be a type of brain-specific diabetes. Curcumin improves cerebral glucose uptake in APPswe/PS 1 dE9 double transgenic mice and at the same time treating progressive impairment of glucose utilization and insulin signal caused by AD. The mechanism of action may be to improve spatial learning and memory to some extent by increasing glucose metabolism and improving damaged insulin signal pathways in the brain [95, 96].

#### 2.2.4 Parkinson’s disease (PD)

PD is a neurodegenerative disorder with the feature of dopaminergic neurons loss in the substantia nigra, abnormal accumulation and aggregation of the pre-synaptic protein a-synuclein in the dopaminergic neurons.

The neuroprotective role of curcumin against Parkinson is explicit because of its prominent antioxidant potential in vivo [97, 98]. Indeed, curcumin treatment could restore GSH levels, prevent protein oxidation and preserve the activity of mitochondrial complex I, which is usually impaired by the loss of GSH. By building dynamic modeling of curcumin-mediated neuroprotection, curcumin could increase the composition of GSH through augmenting the γ-GCL transcription and could prevent GSH depletion-mediated mitochondrial injury in PD [99].

Replenishing striatal dopamine is one of the pharmacological approaches in PD. By the results of tyrosine hydroxylase (TH) immunoreactivity in the substantia nigra and caudoputamen, curcumin treatment increased the expression of α7-nicotinic acetylcholine receptor (α7-nAChR) and had a neuroprotective effect on dopaminergic neurons [100].

Curcumin could afford neuroprotection and inhibit the aggregation of a-synuclein in vitro and in vivo. In the glutathione system (GSH, GSSG and redox ratio), curcumin could prevent the deposition of iron in the dopaminergic neurons, which exert a significant improvement effect. The data analysis of gene and protein activity of a-synuclein also showed that curcumin could prevent a-synuclein aggregates in the dopaminergic neurons [101]. Studies have shown that curcumin has an effect in preventing mitochondrial HKI release and ROS enhancement induced by α-synuclein fibrillation products [102]. The appearance of α-synuclein positive intracyto-plasmic inclusions is the pathological feature of PD. Curcumin could efficiently decrease the pathological accumulation of missense mutation A53T α-synuclein through the mTOR/p70S6K signal pathway, thus reducing the toxic effect on neurons [103]. PD-related neuronal lesions are common in clinical practice. The neuroprotective effect of curcumin was beneficial to the treatment of PD. This study found behavioral manifestations of PD rats were effectively ameliorated by the treatment of curcumin. Curcumin could effectively alleviate the 6-OHDA-induced hippocampal damage, which underlying mechanism was related to the activation of BDNF/TrkB-dependent pathway for accelerating neural regeneration of hippocampal tissue [104]. PINK1 plays an important role in controlling mitochondrial quality via removing dysfunctional mitochondria, whose mutations usually led to early-onset PD with autosomal recessive inheritance, which is documented in a cellular model of PD via siRNA-mediated knock down of PINK1. The cell viability, MMP, mitochondrial respiration and ATP production were significantly decreased. After curcumin pretreatment, apoptosis was significantly decreased, and cell viability was reduced, meanwhile MMP and mitochondrial respiration obviously increased [105].

#### 2.2.5 Depression

As a neuropsychiatric disease, depression is related to a wide range of disruptions in neuronal plasticity all over the brain. Curcumin has been shown to have therapeutic effects on depressive-like disorders.

It was reported that inflammation is associated with the pathophysiology of depression. The antidepressant effects of curcumin are due to its powerful anti-inflammatory effects. Studies showed that the stressed-induced P2X7R/NLRP3 inflammasome axis activation and the transformation from pro-IL-1β to mature IL-1β were inhibited by curcumin. Curcumin supplementation also improves the stress-induced activation of indolamine-2, 3-dioxygenase (IDO) and increase the kynurenine/tryptophan ratio [106]. During post-stroke depression (PSD), curcumin could block the Ca^2+^ accumulation in microglia and repress the neuro-inflammation response mediated by Ca^2+^ channel via the inhibition of P2X7 receptor [107]. The long-term administration of curcumin before stress significantly reduced over-expression of the proinflammatory cytokine IL-1β and phosphorylated-p38 MAPK. What's more, curcumin could inhibited the neuronal apoptosis in the ventromedial prefrontal cortex induced by IL-1β [108]. Curcumin treatment decreased the expression of oxidative stress marker proteins Nox2, 4-HNE and MDA and increased the activity of CAT antioxidant enzyme, thus alleviating oxidative stress. In addition, curcumin increased NQO-1 and HO-1 mRNA expression and activated Nrf2-ARE signal pathway. Curcumin increased the ratio of pCREB/Cyclic AMP response element-binding protein(CREB)and synaptic-related protein [109]. Curcumin increased hippocampal neurogenesis in chronic stressed rats and could reverse or protect hippocampal neurons from further damage caused by depression through the up-regulation of 5-HT1A receptors and brain-derived neurotrophic factor (BDNF), which are two molecules involved in hippocampal neurogenesis [110]. Studies have shown that the neurotrophic effects of antidepressants by increasing neurogenesis may be the cause in treating major depressive disorder. Based on the above findings, hippocampal BDNF contributes to the pathophysiology of depression and maybe a key protein target to curcumin's antidepressant effects [111]. Amygdala is a key structure deemed to be related to depression. Taking curcumin prophylactically could obviously prevent this neuronal dysregulation and depressive-like behaviors caused by chronic, unpredictable, mild, stress (CUMS), and ultra-structural changes was found in neurons within the lateral amygdala (LA) at the same time. The potential antidepressant mechanisms of curcumin may be related to the neuroprotective ability of modulating synaptic plasticity [112]. Studies suggested that curcumin's effect on depression may be mediated by the monoamine neurotransmitter pathway [113]. Curcumin treatment of chronic systolic injury (CCI) in depressive-like mice may be mediated by the supraspinal serotonergic system and downstream GABA_A_ receptor. The antidepression of curcumin was separated from its paralleled antinociception in the context of mononeuropathy [114]. The specific pharmacological action of curcumin against AD, PD and depression and its molecular mechanism were summarized in Table 2.

**Table 2:** Pharmacological mechanism of Curcumin on Neurological disorders.

Disease	Research object	in vitro in vivo	administration route	Mechanism of action	Results	Refs
**AD and PD**	Protein tau	in vitro	-	↓the aggregation and oligomerization of tau	Inhibited the aggregation of tau and dissolved the tau aggregates	[88]
**AD**	N2a/WT cells, N2a/ APP695swe cell and six-month-old APP/PS1 double transgenic mice	in vitro in vivo	p.o.	↓Caveolin-1↓ GSK-3β	Attenuate the hyper-phosphorylation of Tau	[89]
**AD**	SH-SY5Y (human neuroblastoma cells) and HEK293 (human kidney cells)	in vitro	-	↑ERβ directly effects on the upstream factors of the NFκB signaling pathway	Notably reduce Aβ levels and inhibits BACE1gene expression	[90]
**AD**	Adult male and female APPswe/PS1dE9 mice	in vivo	i.v.	↓amyloid deposition ↓Aβ aggregation ↓soluble Aβ40 ↑ oluble Aβ42	Reverses existing amyloid pathology and associated neurotoxicity	[91]
AD	Tg2576 mice (B6; SJLTg (APPSWE) 2576Kh) with the APP Swe transgene); THP-1 cells	in vitro in vivo	p.o.	↓ CD33↑TREM2 ↑ TyroBP ↓miR-155↓genes characteristic of toxic pro-inflammatory M1 microglia (CD11b, iNOS, COX-2, IL-1β)	Stimulate phagocytic clearance of amyloid while restore neuro-inflammatory networks	[92]
**AD**	Amyloid-β (Aβ1 -40) induced AD rat models	in vivo	i.p.	↓GFAP mRNA and the number of GFAP positive cells ↓AS activity	Improves the spatial memory disorders in Aβ1-40-induced rats	[93]
**AD**	APPswe/PS1 dE9 double transgenic mice	in vivo	i.g.	↑ insulin like growth factor (IGF)-1R, IRS-2, PI3K, p-PI3K, Akt and p-Akt protein expression ↓ IR and IRS-1	Improve spatial learning and memory by increasing glucose metabolism and ameliorating the impaired insulin signalling pathways in the brain	[95,96]
**PD**	An PD mice model induced by rotenone	in vivo	p.o.	↑mitochondrial enzyme complex activities ↓acetylcholine esterase enzyme level ↑activities of antioxidant enzymes	Improve behavioral alterations and have antioxidant potential in vivo.	[97]
PD	Transgenic fly lines that express wild-type human synuclein (h-αS)	in vivo	p.o.	↓lipid peroxidation ↓protein carbonyl content↓mean gray scale values	Improve the loss of activity pattern, reduce the oxidative stress and apoptosis, and prolong lifespan to a certain extent.	[98]
**PD**	Adult male C57BL/6 mice; Mouse brain and the 1RB3AN27 (N27) rat dopaminergic neuronal cell line	in vitro in vivo	i.p.	↑GSH levels ↑ γ-GCL gene expression ↑ formation of the EpRE complex and the AP1 transcription factor	Protects mouse brain against GSH depletion-mediated oxidative stress in vivo and in vitro	[99]
**PD**	A 6-Hydroxydopmine-Induced Rat Model of PD	in vivo	p.o.	↑the function of α7-nAChRs expressed	Neuroprotective effect via an α7-nAChR-mediated mechanism.	[100]
**PD**	A rat model of PD induced by LPS	in vivo	i.p.	↓transcription factor NFjB ↓proinflammatory cytokines (TNF-α, IL-1β, and IL-1α) ↓inducible nitric oxide synthase (iNOS)↓ regulating molecules of the intrinsic apoptotic pathway (Bax, Bcl-2, Caspase 3 and Caspase 9)	Modulate the aggregation of a-synuclein in vitro and in vivo.	[101]
**PD**	Male, albino, NMRI rats brain mitochondria	in vitro	-	Preventing mitochondrial HKI release and ROS enhancement induced by α-synuclein fibrillation products	Ameliorate neurodegenerative disorders in PD.	[102]
PD	SH-SY5Y cells	in vitro	-	↓phosphor-mTOR and phosphor-p70S6K↓the accumulation of A53T α-synuclein	Reduce the accumulation of A53T α-synuclein through the mTOR/ p70S6K signaling and recovery of macroautophagy	[103]
**PD**	6-hydroxydopamine (6-OHDA)-PD rat model	in vivo	p.o.	↑ hippocampal brain derived neurotrophic factor (BDNF), ↑TrkB, ↑phospha-tidylinositide 3-kinases (PI3K)	Promoting neural regeneration of hippocampal tissue	[104]
**PD**	SH-SY5Y neuroblastoma cells knocked down of PINK1 via siRNA	in vitro	-	↑ cell viability and maximal respiration ↑mitochondrial membrane potential (MMP)↓apoptosis	Prevent mitochondrial dysfunction and apoptosis in PINK1-deficient cells.	[105]
**Depression**	Chronic unpredictable mild stress (CUMS)-induced depression rat model	in vivo	p.o.	↓NF-κB P65↑IkB↓ IL-1β, IL-6 and TNF-α↓P2X7R, NLRP3, and Caspase-1 P20↓pro-IL-1β and mature-IL-1β↓indolamine-2, 3-dioxygenase (IDO) expression↓KYN content and the KYN/TRP ratio↑5-HT content	Alleviate depression by inhibiting the NLRP3 inflammasome and kynurenine pathway.	[106]
**post-stroke depression (PSD)**	SD rats of PSD model	in vivo	p.o.	↓Ca^2+^channel ↓P2X7R ↓TNF-α ↓IL-1β	Block Ca^2+^ accumulation and neuroinfammation by inhibit P2X7R activity	[107]
**Depression**	CUMS-induced depression rat model	in vivo	i.p.	↓Iba-1↓GFAP↓IL-1β ↓TUNEL positive cell ↑NeuN positive cell	Alleviated depression-like behaviors, expression of the proinflammatory cytokine interleukin-1β (IL-1β) and inhibited neuronal apoptosis within neurons of the ventromedial prefrontal cortex (vmPFC)	[108]
**Depression**	CUMS-induced depression rat model	in vivo	p.o.	↓oxidative stress markers (Nox2, 4-HNE, and MDA) ↑ activity of CAT ↑mRNA expression of NQO-1 and HO-1	Curcumin could alleviate the oxidative stress via Nrf2-ARE signaling pathway to improve depressive-like state.	[109]
**Depression**	Adult rats exposed to a regime of chronic stress	in vivo	p.o.	↑5-HT1A mRNA and BDNF protein levels	Increased hippocampal neurogenesis in chronically stressed rats, prevented the stress-induced decrease in 5-HT1A mRNA and BDNF protein levels in the hippocampal subfields	[110]
**Depression**	Adult male Wistar Kyoto (WKY) rat, a putative model of depression	in vivo	i.p.	Reduction of immobility in the forced swim test (FST); increase in hippocampal brain derived neurotrophic factor (BDNF)	Antidepressant-like effect by increased neurotrophic activity	[111]
Depression	CUMS-induced depression rat model	in vivo	i.p.	↑expression of synapse-associated proteins (such as brain-derived neurotrophic factor, PSD-95 and synaptophysin)and LA synaptophysin	Neuroprotection and antidepressant-like effects in the CUMS induced depression model.	[112]
**Depression**	Olfactory bulbectomy and forced swimming test models of depression in male albino rats	in vivo	p.o.	↑serotonin, dopamine, and noradrenaline ↓3, 4-dihydroxyphenylacetic acid and 5-hydro-xyindoleacetic acid	Exert antidepressant activity in the olfactory bulbectomy and forced swim test model of depression through monoaminergic neurotransmitter pathway	[113]

### 2.3 Inflammatory disease

#### 2.3.1 Rheumatoid Arthritis

Rheumatoid arthritis (RA) is a kind of chronic systemic inflammatory disease characterized by synovial inflammation and joint disability. Immune cells (including T iymphocyte, B iymphocyte and macrophages) infiltrate synovial tissues and increase the release of pro-inflammatory cytokines have participated in the progression of RA. Besides, fibroblast-like synoviocytes (FLS) proliferate and increase the level of IL-6, IL-8, and COX-2 as well as MMPs, which contribute to joint destruction. Curcumin have shown strong anti-inflammatory activity and could induce obvious decrease in cell viability and then apoptosis in FLS [115]. In vitro study shows that curcumin suppressed the degradation of IκBα and decreased the level of COX-2 and induced macrophage apoptosis with strong pharmacological effect on reducing the inflammatory response caused by macrophages [116]. In DBA/1 J mice, curcumin could reduce the severity of collagen-induced arthritis (CIA) along with the decrease of B cell-activating factor BAFF production induced by IFNγ. Further studies showed that curcumin could inhibit STAT1 phosphorylation and nuclear translocation, suggesting that curcumin might inhibit BAFF expression by negatively interfering STAT1 signaling [117]. Recently, mTOR has been reported to be a new therapy target for RA. Curcumin inhibited the increase of IL-1β, TNF-α, MMP-1, MMP-3 and other proinflammatory cytokines in CIA rats. It alleviates CIA inflammation and synovial hyperplasia through the mTOR pathway [118]. Curcumin could suppress chondrocyte apoptosis by activating autophagy and promote chondrogenesis of MSC-like progenitor cells and inhibiting the expression of pro-inflammatory cytokines to establish a suitable microenvironment [119, 120]. Through GC/TOF-MS-based metabolomic investigation on FLS, the level of some metabolites including glycine, citrulline, arachidonic acid and saturated fatty acids was restored after treatment of curcumin. These results suggested that these metabolites may be potential targets in the effect of curcumin on preventing joint inflammation [121]. By increasing the secretion of somatostatin in small intestines, oral administration of curcumin could show significant anti-inflammatory effects through cAMP/PKA and Ca^2+^/CaMKII signal pathways [122].

#### 2.3.2 Osteoarthritis

Osteoarthritis (OA) is a common form of degenerative arthritis. The experimental results show that curcumin could down-regulate the expression of NF-κB targets including COX-2 and MMP-9 and inhibit IL-1β induced stimulation of upstream protein kinase B Akt. Therefore, curcumin has therapeutic potential for OA via IL-1β/TNF-α catabolic signal pathway which is mediated by NF-κB in chondrocytes [123]. TLR4 is an important entry point in the research of innate immunity, and its signal transduction ways cover MyD88 dependent and nondependent pathways. Curcumin could interdict TLR4/NF-κB signal pathway and alleviate inflammation to prevent knee wound on monosodium iodoacetate (MIA)-induced OA rats [124]. Curcumin could suppress the PERK-eIF2α-CHOP axis of the Endoplasmic reticulum (ER) stress response by activating the expression of silent information regulator factor 2-related enzyme 1 (SIRT1) in tert-Butyl hydroperoxide-(TBHP-) induced rat chondrocytes [125]. Curcumin could also inhibit the production of catabolic mediators by chondrocytes, particularly S-glycosaminoglycans (GAG) from human cartilage explants [126]. Curcumin could inhibit inflammation in osteoarthritic environment by regulating NF-κB cartilage-specific proteins (collagen II, CSPG, Sox9) coupling and maintain homeostasis in OA by controlling chondrocyte survival [127]. Curcumin showed a chondroprotective effect on IL-1β induced primary chondrocytes through the extracellular signal-regulated kinases 1/2 (ERK1/2)-induced autophagy in vitro [128]. In spontaneous and surgically induced OA mice model, curcumin accelerated cells autophagy through Akt/mTOR pathway, which presented as the increased expression of LC3 and Beclin1, as well as inhibited apoptosis (cleaved caspase-3 and Bax/Bcl2) and the degradation of cartilage matrix (MMP13, ADAMTS-5, COL2A1, and aggrecan) [4]. After curcumin treatment, the exosomes derived from mesenchymal stem cells (MSCs) could restore the expression level of miR-143 and miR-124 as well as NF-κB and ROCK1 in OA cells by decreasing the methylation of DNA in miR-143 and miR-124 promoters. In addition, the binding sites of miR-143 and miR-124 were confirmed to exist in the 3’ UTRs in NF-κB and ROCK1, as well as the up-regulation level of miR-143 and miR-124 could significantly restore the expression of NF-κB and ROCK1 in cells and mouse OA models treated with exosomes [129]. External use of curcumin nanoparticles could also alleviate OA-related pain compared with oral curcumin by decreasing tactile hypersensitivity and improving locomotor behavior in a mouse model of destabilization of the medial meniscus (DMM) [3].

#### 2.3.3 Inflammatory bowel diseases (IBDs)

Inflammatory bowel disease (IBD) is characterized by chronic inflammation located in the gastrointestinal tract, whose incidence is rising, especially in young people.

In IBD mouse model, curcumin activates α-catenin, inhibits immune response, increases exogenous metabolism, and alleviates inflammation. Curcumin also down-regulated key transcription factors and other regulatory molecules involved in inflammatory activation, such as ERK, FN1, TNFSF12 and PI3K complexes [130]. Curcumin inhibited the activation of NLRP3 inflammasome by inhibiting DSS induced K+ efflux, intracellular ROS production and the release of cathepsin B. The downregulation of IL-1β secretion, caspase-1 activation and apoptosis associated speck-like protein (ASC) specks were also convictive signs [131]. Through colonic mucosal biopsies and colonic myofibroblasts (CMF) of children and adults with IBD, curcumin suppressed p38 MAPK activation and the level of MMP-3 and IL-1β, as well as enhanced IL-10 [132]. Curcumin could accelerate the recovery of impaired colonic mucosa by potentially the activation of (dendritic cells) DCs to enhance the inhibiting effect of CD4^+^CD25^+^Foxp3^+^T cells (Treg cells), which had an important effect on controlling self-tolerance and keeping immune homeostasis [133]. IFN-γ has been reported to have an effect on epithelial integrity and promoting barrier dysfunction and epithelial permeability by multiple mechanisms. Studies have shown that curcumin suppressed IFN-induced gene transcription in human and mouse colonocytes [134]. Mitochondrial dysfunction also plays a role in the pathogenesis of IBD. In 2, 4, 6-trinitroben-zene sulfonic acid (TNBS)-induced chronic colitis mice model, curcumin inhibited ROS generation intra and extra mitochondrion, balanced aconitase/fumarase and MDA/GSH ratios, and restored the connection between mitochondria [135].

### 2.4 Cardiovascular diseases

#### 2.4.1 Ischemia-reperfusion (IR)

Ischemia-reperfusion (IR) injury has always been an intricate and complicated situation in research and clinical practice. It’s reported that curcumin could suppress oxidant damage and mitochondrial dysfunction induced by IR and play a protective role in damaged isolated hearts [9]. The TGFβ1/Smad pathway activated by oxidative stress is associated with the collagen synthesis in heart,which could be suppressed by curcumin in maladaptive cardiac repair [136].

In rat hearts after IR injury, curcumin improved cardiac function, decreased infarct size and increased lactate dehydrogenase levels. Curcumin could also increase H9c2 cell viability, inhibit apoptotic and regulate certain biochemical parameters, such as anti-apoptotic protein Bcl-2, proapoptotic protein Bax and AcSOD2, SIRT3, SOD (serum superoxide dismutase) and GSH-Px activity, and MDA (malondialdehyde) content [127]. Through JAK2/STAT3 signal pathway, curcumin might inhibit myocardium apoptosis by reducing oxidative damage and decrease the risk of coronary heart disease [137], as well as delivering a survival signal to the myocardium thereby alleviating IR injury [138]. In rat model of isoprenaline induced myocardial ischemia, curcumin protected myocardium against ischemic damage, which may be associated with its powerful antioxidant properties as well as the inhibiting effects on xanthine dehydrogenase/ xanthine oxidase (XD/XO) conversion and the production of superoxide anion [139]. By increasing the level of miR-7a/b and decreasing transcription factor specific protein 1 (SP1) expression, curcumin could prevent hypoxia-induced cardiac myocytes from apoptosis [140].

#### 2.4.2 Cardiac hypertrophy and fibrosis

Hemodynamic excess load in the heart would induce maladaptive hypertrophy of cardiomyocytes. Evidence indicated that p300, an intrinsic histone acetyltransferase has an important effect in this pathological process. Sunagawa et al illustrated that curcumin could suppress hypertension-induced increase of posterior wall thickness and LV mass index. Further research proves that hypertension-induced increases in myocardial cell diameter, perivascular fibrosis. The transcriptions of the hypertrophy-response gene (GATA4) were also be inhibited by curcumin [141]. By upregulating expression of Na^+^/Ca^2+^ exchanger (NCX) after transverse aortic constriction (TAC), curcumin could inhibit cardiac hypertrophy, improve cardiac systolic/diastolic function, and preserve vascular endothelium cells [142].

Curcumin could balance degradation and synthesis of collagens as well as inhibit myocardial fibrosis by adjusting the levels of angiotensin II (Ang II) receptors and angiotensin-converting enzyme 2 (ACE2) to improve cardiac function [143]. Ang II-mediated cardiomyocyte growth could be suppressed by curcumin via inhibiting lipoprotein (ox-LDL) receptor-1 (LOX-1) and Ang II type 1 receptor (AT1R) expression and restoring the heightened condition of intracellular redox [144, 145], as well as down-regulating the level of SIRT1 after myocardial infarction (MI). In isoproterenol-induced cardiac hypertrophy and fibrosis rat model, curcumin played an obvious therapeutic role via mammalian target of rapamycin (mTOR)/autophagy axis [146]. Besides, Curcumin could inhibit the expression of NF-κB-mediated nucleotide-binding oligomerization domain-like receptor protein 3 (NLRP3) to alleviate hypertension, vascular inflammation, and vascular remodeling [147].

#### 2.4.3 Other cardiovascular diseases

Coronary microembolization (CME) -induced local myocardial inflammation and myocardial apoptosis and cardiac dysfunctions could be alleviated by curcumin in some degree via Toll-like receptor 4 (TLR4)/ myeloid differentiation primary response 88 (MyD88)/NF-κB signal pathway [148]. Toll-like receptor 2 (TLR2), an important mediator in innate immune system, is associated with myocardial infarction and could be selectively suppressed by curcumin, thus curcumin may prevent and treat myocardial infarction [149]. In rats with sepsis induced by cecal ligation and puncture (CLP), curcumin obviously strengthens myocardial contractility by increasing the Ejection Fraction (EF) and Fractional Shortening (FS). Inflammation and injury in myocardial cells of sepsis induced by CLP could also be alleviated [150]. 14-3-3γ is a member of the 14-3-3 protein family members, which take part in regulating diverse biological processes, including cell division, signal transduction, and apoptosis by interaction with their effectors. Among them, it was reported that 14-3-3γ was associated with myocardial injury and protection. Curcumin could increase the expression of 14-3-3γ, thus promoting the translocation of Bcl-2 to mitochondria, restraining oxidative stress, and enhancing mitochondrial function to prevent the myocardium from Dox-induced injury [151].

### 2.5 Metabolic diseases

#### 2.5.1 Obesity

Obesity, characterized as the pathological hyperplasia or/and hypertrophy of adipose tissue, is one of the major contributing factors of metabolic syndrome. Curcumin could promote preadipocyte apoptosis as well as suppress adipocyte differentiation, and thus inhibiting adipogenesis [152]. An in vitro study reported that curcumin inhibited mitotic clonal expansion (MCE), reduced the activation of peroxisome proliferator-activated receptors (PPARγ) and CCAAT enhancer binding proteins (C/EBPα), as well as the lipid accumulation in 3T3-L1 adipocytes [153]. Inhibiting the level of FAS, curcumin could suppress adipocyte differentiation and lipid accumulation [154]. In liver and fat tissues, 11b-hydroxysteroid dehydrogenase 1 (11b-HSD1) could activate the expression of glucocorticoid partly and aggravate metabolic syndrome. In the study, curcumin played an obvious role in inhibiting the production of 11b-HSD1 in both human and rat cells [155]. Curcumin influenced energy expenditure mediated by brown adipose tissue (BAT) and inflammation of white adipose tissue (WAT). High-fat diet induced obesity model mice studies illustrated that curcumin could inhibit the increased expression of uncoupling protein 1 (UCP1) in BAT, intervene the reduced of macrophage infiltration and altered macrophage functional polarity in WAT, of high-fat diet induced obesity mice [156]. Fetuin-A, a protein synthesized in liver and excreted into bloodstream, is related to the physiopathologic mechanism of metabolic disorders including visceral obesity. Curcumin treatment may have potential in down-regulating triglycerides in liver and fetuin-A levels in serum, which may involve in the salutary effects in the pathogenesis of obesity [157]. Hypoxia caused by excessive expansion of white adipose tissue damage would impair normal metabolism, which could be inhibited by curcumin via protecting mitochondria and alleviating inflammation [158]. Curcumin could inhibit adipose tissue growth via antiangiogenic effect, which was demonstrated by the decreased vascular endothelial growth factor (VEGF) and its receptor VEGFR-2 expression. The fatty acid esterification was also relieved by curcumin via up-regulating 5#AMP-activated protein kinase phosphorylation as well as carnitine palmitoyl-transferase-1 expression, and down-regulating glycerol-3-phosphate acyl transferase-1. In addition, curcumin obviously reduced serum cholesterol and PPARγ, as well as CCAAT/enhancer binding protein α level, which are important transcription factors in the formation of adipose tissue [159]. The inhibiting effect of curcumin on lipogeneis in liver is related to suppressing the expression of carbohydrate response element binding protein (ChREBP) and SREBP-1c [160].

#### 2.5.2 Diabetes mellitus

Diabetes mellitus is a cluster of metabolic disorders because of absolute or relative insufficient insulin secretion and less sensitivity of target cells to insulin, which is characterized by hyperglycemia. Type 1 diabetes (T1DM) and Type 2 diabetes (T2DM) are the most common types of diabetes. The primary clinical manifestations of diabetes are polydipsia, polyuria, weight loss, high blood sugar and glucose in urine. Moreover, if not treated timely, diabetes would cause pathological effects on other parts of the body.

T1DM is characterized by an absolute insulin deficiency and is basically caused by immune-mediated beta cells destruction in pancreas. During autoimmune diabetes, curcumin could regulate the harmful proliferation in T lymphocyte response and IFN-γ production by influencing T-bet, an important transcription factor in the effect of pro-inflammatory Th1 lymphocyte differentiation. Besides, curcumin inhibited the dendritic cells-induced T cell activation via down-regulating pro-inflammatory cytokines, NO and the expression of surface coestimulatory molecules, which contributed to a total reduce of antigen presenting cytoactive [161]. In the streptozotocin-induced T1DM model of rats, curcumin prevented kidney damage through regulating Nrf2, NF-κB and NADPH oxidase, as well as by protein kinase CβII (PKCβII)/p^66^Shc axis [162].

T2DM accounts for more than 90% of diabetics, which is characterized by relative insulin deficiency and insulin resistance. Curcumin could obviously decrease blood glucose and glycosylated hemoglobin (HbA1c) levels, as well as improve insulin resistance in homeostasis model appraisal and elevate plasma insulin content [163]. In mitochondria from the liver and kidneys of diabetic mice, hyperglycaemia changes oxygen consumption rate, NO synthesis and up-regulates the level of thiobarbituric acid-reactive substances (TBARS). Nevertheless, curcumin may show potentially beneficial influences on these alterations [153, 154]. In the skeletal muscle of rats with vast fructose supplement, curcumin alleviated insulin resistance by reducing Insulin receptor substrate (IRS-1) serine phosphorylation and IRS-1 tyrosine phosphorylation. Curcumin could regulate Sterol regulatory element-binding proteins (SREBPs) target genes and metabolism relative genes. Curcumin would be associated with the downregulation of free fatty acid (FFA) and TNF-α in serum and have effect on down-regulating lipid level [166, 167]. Curcumin can alleviate diabetes-induced endothelial dysfunction by decreasing the production of superoxide and inhibiting vascular protein kinase C (PKC-bII) [168].

The expression of AMP-activated protein kinase (AMPK), PPARγand NF-κB may be important influencing factors of T2DM complications, which could be regulated by curcumin [169]. Diabetic cardiomyopathy (DCM) is a common complication of T2DM. Curcumin had therapeutic effects on diabetic cardiomyopathy treatment via Sirtuin 1-Foxo1 and PI3K-Akt pathways and suppressed collagen synthesis in rat myocardium by restricting the activation of TGF-β1, canonical Smad signaling and the non-canonical AMPK/p38 MAPK pathway [170, 171]. Also, curcumin suppressed high mobility group box 1 (HMGB1) signal pathway in both vivo and invitro and play a protect effect on myocardial injury [172]. Curcumin may have potential effect on improving the development of diabetic nephropathy by suppressing Protein kinase C (PKC) -α and PKC-β1 activity-ERK1/2 pathway, as well as by partly adjusting the functional connections between cav-1 (a major multifunctional scaf-folding protein of caveolae) and ROS in vivo [173, 174]. In addition, curcumin could mediate autophagy and inhibit podocyte EMT via the PI3k/Akt/mTOR pathway [5]. Diabetes-induced endothelial dysfunctions could be ameliorated by curcumin via reducing leukocyte-endothelium interaction, decreasing the overproduction of ROS and suppressing ICAM-1 and NOX2 expression; Moreover, curcumin could also decrease the expression of IL-6, MCP-1, TNF-*α*, glucose, HbA1, and oxidative stress in blood, thereby exert the improvement of diabetic vascular inflammation [175-177]. Curcumin may prevent diabetic testicular injury via inhibiting testicular apoptosis by regulating apoptotic proteins [178]. In diabetic pathophysiology induced splenic damage, curcumin could prevent cells against inflammatory damage by modulating the level of inflammatory cytokines, chemokines, adhesion molecules and the translocation of NFκB into the nucleus [179]. In the liver, curcumin could alleviate the development of T2DM by anti-apoptotic activity and regulating phosphatidylinositol 3-hydroxy kinase/protein kinase B signal pathway [180].

At present, the majority active mechanism study of curcumin is in vitro; the study in vivo is more needed and meaningful to illustrate the treatment mechanism. Though amounts of research reported that curcumin has excellent pharmacological activity, the druggability issue blocked its progress of clinical application.

**Figure 5.**
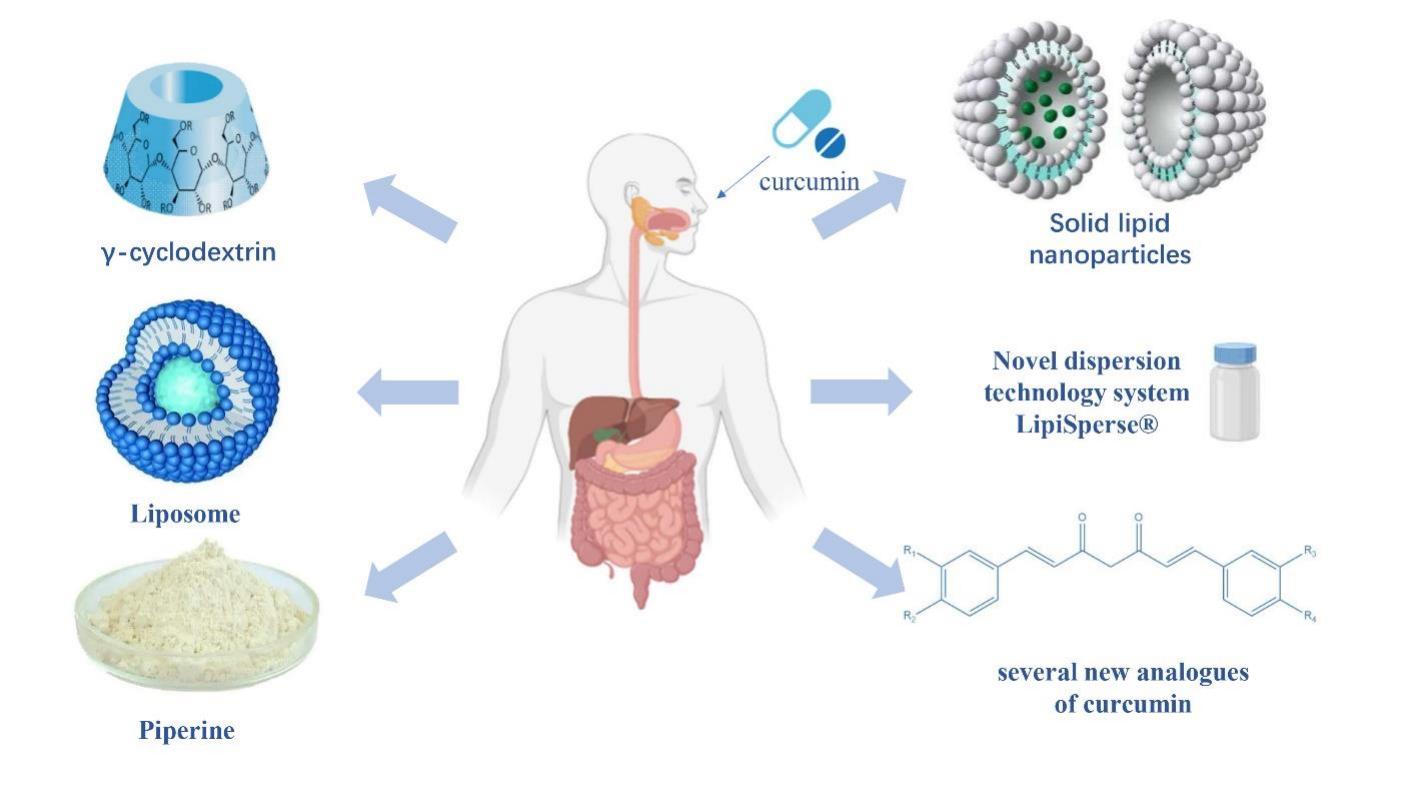
Methods for improving bioavailability of curcumin.

## 3. The main problems and research progress of curcumin in clinical application

### 3.1 Poor bioavailability of curcumin

Despite so many scientific experiments that have shown its beneficial effects for cancer, neurological disorders, inflammatory diseases, cardiovascular diseases and metabolic diseases, the potential of curcumin seems to be limited by its poor absorption. Numerous pharmaco-kinetics studies indicated that low bioavailability of curcumin is due to: (a) quick elimination and clearance; (b) poor gastrointestinal absorption; (c) inactivity of metabolites. Literature reported that ninety percent of curcumin was metabolized within 30 minutes through detecting the suspensions of separated rat hepatocytes or liver microsomes [181]. In order to find out the metabolism of curcumin, researchers conducted a series of experiments. The results indicated that curcumin metabolites including curcumin glucuronide, curcumin sulfate, tetrahydrocurcumin and hexahydrocurcumin were found in intestinal, hepatic microsomes and hepatic cytosol from humans and rats. Moreover, it is interesting to found that the degree of curcumin conjugation in intestinal tracts from humans was much greater than those from rats, but curcumin conjugation in hepatic fractions from humans was less extensive than those from rats [182]. In a human clinical trial, the plasma curcumin level was only 11.1 nmol/L via oral administration of 3.6 g curcumin in an hour [183]. Another trial study of curcumin given orally indicated that most curcumin was detected in feces in an unmetabolized form rather than detected in blood or urine [12, 184]. In addition, the other little part of absorbed curcumin and its metabolites are quickly converted into water soluble metabolites, including glucuronides and sulfates [185, 186]. In rats, a little bit of dissociative curcumin after being taken orally is detected, while most metabolites of curcumin are discovered as conjugates (curcumin glucuronide, curcumin sulfate) in blood [187]. Similar results in human trials show that glucuronide conjugates and sulfate conjugates are detected, but free curcumin is almost undetected after supplying curcumin per oral dose [188].

The bioavailability of orally taken curcumin in the intestine is almost zero. Only the conjugated and reductive metabolites of curcumin are partially absorbed in the intestine. Therefore, most pharmacological effects of curcumin in tissues except the gastrointestinal tract may be due to curcumin metabolites [10]. In a pilot trial on the sick with hepatic metastases from colorectal cancer, oral administration of curcumin was poorly available in liver tissue, but its metabolic reduction products were found. Moreover, this study compared the oxidative DNA fluctuation in healthy and malignant human liver tissue after oral administration of curcumin. The result suggested that afford sufficient curcumin of hepatic required levels to exert pharmacological activity maybe not achievable in humans [11]. Another clinical trial also found that despite taking the maximum restricted oral dose of curcumin, it still could not be detected in any subject plasma and the expression of HO-1 in peripheral blood mononuclear cells is not affected at all [189].

### 3.2 Primary methods to improve the bioavailability of curcumin

For the past decades, varieties of methods have been conducted to improve the solubility and bioavailability of curcumin, such as nanomaterials drug delivery systems, different kinds of adjuvants (piperine‚ quercetin or silibinin), curcumin structural analogues, bioconjugates of curcumin with turmeric oil or alanine and chemical complex of curcumin with phospholipids, proteins or polysaccharides [190], the specific methods were shown in Figure 5. Nanomaterials as an emerging effective method has been widely used in drug delivery system for its advantage of increase half-life, protect drugs from degradation, enhance biocompatibility, solubility as well as blood circulation time [191, 192]. Nanomaterial drug delivery systems include varieties of dosage forms, such as nanoliposomes, nanoparticles, nanocapsules, nano-crystals, nanospheres, micelles, solid lipid nanoparticles, and dendrimers. As a kind of inclusion material, cyclodextrin can effectively improve the water dispersion and bioavailability of drug by forming inclusion compound with it [193]. Researches have demonstrated that γ-cyclodextrin curcumin formulation could markedly further the absorption and utilization of curcumin in healthy people [194]. Metal-organic framework is an emerging crystalline porous carrier, which has been widely used in drug delivery due to their wonderful porosity, good loading capacity, and ease of surface modification. A fascinating study successfully synthesized a safe and biodegradable γ-cyclodextrin-metal-organic-framework for effectively improving the physicochemical stability and controlled-release performance of curcumin [195]. Liposome refers to macrovesicles formed by encapsulating drugs in lipid bilayers. As a revolutionizing carrier, liposome has revealed obvious advantages in drug delivery. When curcumin is encapsulated by liposome, it would be able to intravenous dosing and to avoid the trouble of poor oral bioavailability. A review summarized studies in this area and indicated that liposomes could improve antineoplastic and pharmacological activities of curcumin by enhancing drug metabolism and pharmacokinetics (DMPK), pharmaco-dynamics and decreasing the dosage for targeting tumor. Moreover, curcumin incorporation with different adjuvants (vitamin A, folic acid, hyaluronic acid, and silica) encapsulated in liposomal nanoparticles which could improve the sensibility on tumor cells [196]. In recent years, nanoparticles have sprung up as promising drug delivery systems for improving the aqueous solubility and bioavailability of curcumin. Solid lipid nanoparticles (SLNs) are considered as a kind of development potential colloidal carrier system, whose main advantages include making drug be release under targeting and control and preventing incorporated compound from chemical degradation. Curcuminoids loaded solid lipid nanoparticles could strongly reduce the light and oxygen sensitivity of curcuminoids and have significant effects on the mean particle size, the curcuminoid loading capacity. The long-term release of curcumin from curcuminoids loaded (SLNs) can be up to 12 hours according to the Higuchi’s square root model [197]. According to the biological barriers in different diseases [198], kinds of nanomaterials were used to encapsulate curcumin forming nanoparticles, such as poly (lactic-co-glycolic acid) (PLGA) nanoparticles for cancer therapies [199], phenylboronic acid-conjugated chitosan nanoparticles [200], curcumin-loaded hybrid nano-particles (enzyme-targeted peptides, star-shaped polycyclic lipids) [201] and et al. In addition, curcumin-loaded nanocapsules [202], nanocrystals [203], nanosphere [204], and nanomicelles were all attempted by researchers to improve curcumin’s pharmacokinetic parameters and achieved a certain degree of research progress. The emergence of nanomaterials not only improves the bioavailability of curcumin, but also improves the targeting and therapeutic effect of curcumin in different diseases.

**Table 3:** Primary methods to improve the bioavailability of curcumin

Method	Theory	Effect	Ref.
**γ-cyclodextrin curcumin inclusion complexes**	Form inclusion complexes on a molecular basis with liposoluble compounds, thereby increasing water solubility, dispersibility, and absorption.	Significantly improve the absorption of curcuminoids in healthy humans.	[194]
**Metal-organic framework (curcumin encapsulated by γ-cyclodextrin-metal-organic-frameworks)**	A kind of emerging crystalline porous carrier which has excellent porosity, high loading capacity, and ease of surface modification, but exists inherent toxicity and non-biodegradability.	Exhibited improved loading capacity, physicochemical stability as well as controlled-release property in simulated digestion	[195]
**Liposomal curcumin**	Liposome refers to microvesicles formed by encapsulating drugs in lipid bilayers.	Liposomal curcumin formulation has greater growth inhibitory and pro-apoptotic effects on cancer cells.	[196]
**Curcuminoids loaded solid lipid nanoparticles**	A promising colloidal carrier system (made from biodegradable solid lipids exist in the submicron size range)	Making curcumin released under targeting and control and also preventing incorporated compound from chemical degradation.	[197]
**Curcumin encapsulation in functional PLGA nanoparticles**	A non-toxic, biodegradable, and biocompatible polymer; Represents an effective strategy to deliver a drug to a tumor site.	Encapsulation of curcumin in mono-drug PLGA nanoparticles represents an effective tool to overcome its limitations including low bioavailability and poor pharmacokinetics both in vitro and in vivo.	[199]
**Phenylboronic acid-conjugated chitosan nanoparticles**	A non-toxic, biodegradable, and biocompatible polymer; Represents an effective strategy to deliver a drug to a tumor site.	The drug-loaded nanoparticles performed an enhanced growth inhibition in three-dimensional multicellular tumor spheroids	[200]
**Curcumin-loaded hybrid nanoparticles**	Hybrid nanoparticles act as multifunctional drug delivery systems combines the advantages of multiple nanoparticles (with different particle sizes, potentials, and morphologies) for drug delivery, exhibiting collective properties different from those of single nanoparticles.	Effectively improve the bioavailability of curcumin and have potential applications in drug delivery and tumor treament.	[201]
**Curcumin-loaded nanocapsules**	Polymeric nanocapsules, which consist of vesicular nanostructures containing an oily core surrounded by a polymeric wall.	curcumin-loaded polymeric nanocapsules inhibit tumor growth and decrease tumor weight in rodents is established, regardless of the solid tumor model.	[202]
**Curcumin-loaded nanocrystals**	Drug nanocrystals are generally recognized as a carrier-free submicron colloidal dispersion system consist only of Active Pharmaceutical Ingredients (APIs) and essential stabilizers, the latter of which were used to prevent or reduce the aggregations of pure drug crystals.	Could not rapidly and fully digest in vitro digestion model though it has relatively stable to chemical transformation. Moreover, it had low bioaccessibility.	[203]
**Curcumin-loaded nanoemulsions**	Nanoemulsions are referred to as dispersed systems with≤100 nm droplets. Nanoemulsions are immiscible liquids consisting of oil and water forming a single phase by an emulsifer such as the surfactants and co-surfactants. The combination of these constituents confers high thermodynamics, stability, and other physicochemical properties on the emulsion.	The nanoemulsions have relatively stable to chemical transformation and could rapidly and fully digested in the static in vitro digestion model. Moreover, it had high bioaccessibility to form mixed micelles to solubilize the curcumin.	[203]
**Curcumin-loaded oil bodies**	Curcumin-loaded oil bodies (soy milk)	Relatively stable to chemical transformation; Rapidly and fully digested in vitro digestion model. Moreover, it had high bioaccessibility to form mixed micelles to solubilize the curcumin.	[203]
**Curcumin-loaded nanosphere**	Nanospheres are colloidal drug delivery systems, which act as transport carrier compartments for drugs or other active molecules, with size ranging between 10 and 1000 nm.	The bioavailability was increased in the blood of mice orally fed with curcumin-loaded nanosphere. Besides, the curcumin-loaded nanospheres could also improve the cytotoxicity of the curcumin towards two cancer cell lines including HepG2 and MCF-7.	[204]
**Novel dispersion technology system (LipiSperse®, a mixture of surfactants, polar lipids and solvents)**	LipiSperse® specifically chosen for their ability to embed into the lipophilic crystal structure of the active ingredient, while keeping the hydrophilic head on the surface. This in turn increases the wettability of the crystal, by lowering the surface tension, which allows it to disperse in water.	Curcumin with LipiSperse® delivered significantly higher plasma curcuminoid concentrations compared to the raw curcumin product.	[205]
**Curcumin-piperine combination**	a potent inhibitor of drug metabolism (inhibitor of hepatic and intestinal glucuronidation)	Improve the serum concentration, extent of absorption and bioavailability of curcumin in both rats and humans; reducing oxidative stress burden in obese individuals; significantly improves oxidative and inflammatory status in patients with metabolic syndrome.	[207-209]
**Synthesis of curcumin chemical analogues**	Novel curcumin analogues produced by synthetic chemical modifications	The analogues could decrease the protein B-catenin, Ki-ras, cyclin D1, c-Myc, and ErbB-2 at as low as one eighth the concentration at which curcumin normally has an effect. In addition, the analogues exhibited no toxicities in vivo which may provide effective alternative therapies for the prevention and treatment of some human cancers.	[210]

Novel dispersion technology system has also been put into increasing the bioavailability of curcumin. For example, LipiSperse®, an advanced crystalline coating system that changes the physical properties of the surface of a lipophilic crystal to create a hydrophilic surface. This LipiSperse® technology stops lipophilic ingredients agglomerating in aqueous environments (such as the stomach). Clinical trials have shown that the plasma concentrations of curcumin are obviously increased by the delivery system LipiSperse® when compared to the only curcumin extract supplement [205].

Piperine, as a known inhibitor of drug metabolism, could markedly prevent hepatic and intestinal glucuronidation [206]. Compared with curcumin alone, simultaneous administration of piperine obviously promote the degree of absorption, serum concentration and bioavailability when experimental results are evaluated in rats and healthy human volunteers [207]. Curcuminoid-piperine combination significantly could reduce the balance of serum pro-oxidant-antioxidant balance in a randomized double-blind trial of obese individuals [208]. Other study shows that curcuminoid-piperine coalition markedly improves oxidative and inflammatory state by enhancing SOD activities and reducing concentrations of MDA and plasma C-reactive protein (CRP) in the sick with metabolic syndrome [209].

Synthesis of curcumin chemical analogues could also improve the poor bioavailability,increase the application potential, as well as keep its low toxicity [210]. For the past decades, a huge number of curcumin analogues were designed by researchers in this field. Various functional groups were introduced at different sites (including diaryl moieties, 4-arylidene, C-4 position, and et al) in curcumin. More details can be found in these references [211-213]. The primary methods to improve the bioavailability of curcumin were shown in Table 3.

Finally, it is worth nothing that the effective results of experimental study on pharmacological activity of oral administration curcumin need to be verified due to its poor oral bioavailability. In the future, the related researchers need to notice that the route of administration of curcumin may highly correlate with its pharmacological activity.

## 4. Curcumin clinical application status

Since the discovery of curcumin, research on the activity of curcumin has been a research hotspot in the medical field. Moreover, the clinical trials of curcumin also increased in a progressive manner these years. From now on, the clinical trials of curcumin are primary about cancer [214], AD [215], metabolic syndrome [216], polycystic ovarian syndrome [217] et al. In addition, clinical trials of nanocurcumin [190] and its bioavailability research [218] were also reported. Many clinical trials have shown the role of curcumin in improving diseases, but small number of clinical trials also found that curcumin has no significant benefit to related diseases [219]. The related information about curcumin clinical trials was shown in Table 4. More details of curcumin clinical trials can refer to these references [220-222].

**Table 4:** Clinical application status of curcumin.

Disease	Dose	Duration	administration route	Patients	Results	Ref.
Overweight	80 mg/Day	6 weeks	p.o.	48 obesity girls	Antioxidant effect and prevention of lipid peroxidation in overweight individuals.	[225]
Overweight and obesity	500 mg/Day	10 weeks	p.o.	60 girl adolescents	Positive effects on inflammation and oxidative stress markers	[226]
Metabolic syndrome	80 mg/Day	12 weeks	p.o.	50 Patients	Significantly improved serum triglyceride in Metabolic syndrome patients.	[227]
Nonalcoholic fatty liver disease	80 mg/Day	3 Months	p.o.	84 Patients	Nanocurcumin improves glucose indices, lipids, inflammation, and nesfatin in overweight and obese patients with nonalcoholic fatty liver disease (NAFLD).	[228]
depression and anxiety in diabetic patients with peripheral neuropathy	80 mg/Day	8 weeks	p.o.	80 Patients	Nano-curcumin can significantly decrease the anxiety and depression level of patients	[229]
Type 2 diabetes	1500 mg*3/Day	10 weeks	p.o.	53 participants with type 2 diabetes	Significant changes in mean weight, body mass index (BMI), waist circumference and fasting blood sugar.	[230]
Alzheimer disease	4 g/Day	6 months	p.o.	35 patients older than 50 years old	Serum Aβ_40_ tended to rise on curcumin, possibly reflecting an ability of curcumin to disaggregate Aβ_40_ deposits in the brain, releasing the Aβ_40_ for circulation and disposal. Improved vitamin E (a plasma antioxidants) of Alzheimer disease patients	[231]
Schizophrenia	90 mg/Day	12 weeks	p.o.	12 Patients	Significantly improved working memory, cognitive function, and reduced IL-6 levels.	[232]
Prostate cancer	1440 mg/Day	6 months	p.o.	97 participants	The proportion of patients with prostate-specific antigen progression was significantly lower in the curcumin group than the placebo group	[233]
Cancer	500mg/12h+ 5mg piperine	9 weeks	p.o.	80 Patients	Hematological and biochemical analysis showed no statistical differences between the groups at the end of the trial. But significant differences were observed in hemoglobin, hematocrit, lactic acid dehydrogenase, serum glutamic-oxaloacetic transaminase, and anaplastic lymphoma kinase between the groups.	[234]
Polycystic ovary syndrome	500mg*3/Day	12 weeks	p.o.	67 Patients	Fasting plasma glucose and Dehydroepiandrosterone levels had decreased significantly; non-significant increase in Estradiol levels	[235]
Non-Alcoholic Fatty Liver Disease (NAFLD)	1.5 g/Day	12 weeks	p.o.	52 Patients	A between-group change was not significant after adjustment for multiple testing. After the intervention, there were a lower number of patients with severe fatty liver and metabolic syndrome in the curcumin group compared to the placebo. In conclusion, curcumin offers no additional cardiometabolic benefits to lifestyle intervention in patients with NAFLD.	[236]

## 5. Conclusions

Numerous experimental data in vitro and in vivo have manifested that curcumin have significant pharmacological effects on cancer, inflammatory diseases, neurological disorders, cardiovascular diseases, and metabolic diseases et al because of its multi-target pharmacological actions. These wide ranges of pharmacological activities make it possible for the clinical application of curcumin. Despite the poor bioavailability issues of curcumin, promising approaches including the use of cyclodextrin inclusion compound, liposome microcapsules, drug metabolism blocker (Piperine), novel dispersion technology, solid lipid nanoparticles and the synthesis of curcumin analogues have been conducted to improve its bioavailability. However, further clinical trials and preclinical studies still needed to be carried out to prove the safety and efficacy of curcumin as well as its new dosage forms. In conclusion, this review provides a scientific basis for more profound investigations and clinical applications of curcumin.
